# Supplementary material for: Mutational signatures of DNA mismatch repair deficiency in C. elegans and human cancers
Source: Genome Res. 2018 May;28(5):666–75. doi: 10.1101/gr.226845.117 (PMC5932607; doi:10.1101/gr.226845.117)
Supplement: Supplemental Material [file supp_28_5_666__index.html]

Mutational signatures of DNA mismatch repair deficiency in C. elegans and human cancers — Mutational signatures of DNA mismatch repair deficiency in C. elegans and human cancers — Supplemental Material 

# Mutational signatures of DNA mismatch repair deficiency in *C. elegans* and human cancers

## Supplemental Material

- Supplemental\_Fig\_S1.pdf
- Supplemental\_Fig\_S2.pdf
- Supplemental\_Fig\_S3.pdf
- Supplemental\_Fig\_S4.pdf
- Supplemental\_Fig\_S5.pdf
- Supplemental\_Fig\_S6.pdf
- Supplemental\_Fig\_S7.pdf
- Supplemental\_data\_analysis.docx
- Supplemental\_Material.docx
