# Supplementary material for: Mutational signatures of DNA mismatch repair deficiency in C. elegans and human cancers
Source: Genome Res. 2018 May;28(5):666–75. doi: 10.1101/gr.226845.117 (PMC5932607; doi:10.1101/gr.226845.117)
Supplement: Supplemental Material [file supp_gr.226845.117_Supplemental_Fig_S1.pdf]

A

T>A (in ATT context)

two adjacent repeats

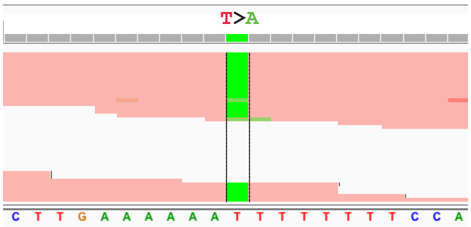

single repeat

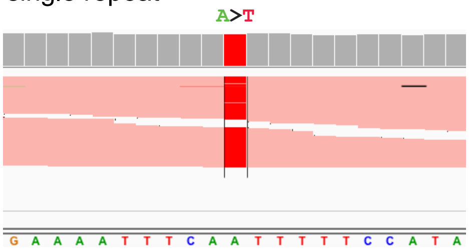

B

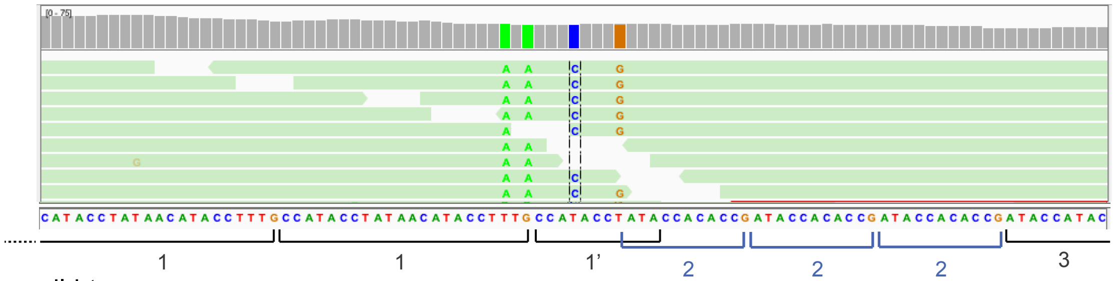

wild-type

1’

(CCATACCTATAACATACCTTTG) 2 CCATACCTATAACATACCTTTG CCATACCTATACCACACCG (ATACCACACCG) 2

1 1 2 2

mlh-1 F20 (CD0134a)

(CCATACCTATAACATACCTTTG) 2 CCATACCTATAACATACCTATA CCACACCGATACCACACCG (ATACCACACCG) 2

1 1* 2 2 2

C

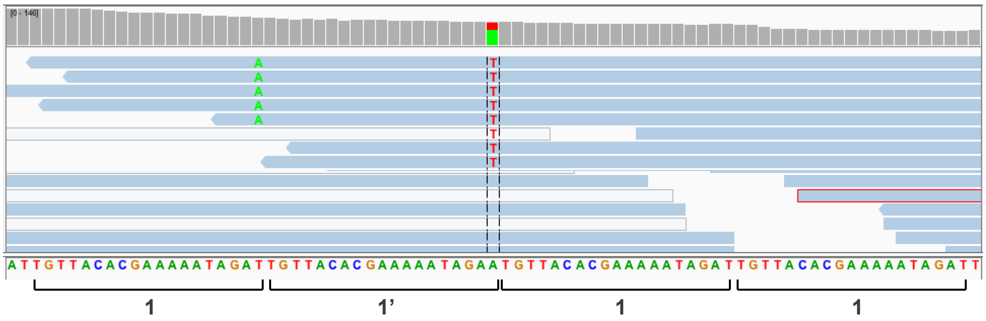

wild-type

(TGTTACACGAAAAATAGAT) n TGTTACACGAAAAATAGAA (TGTTACACGAAAAATAGAT) 2

1 1’ 1

mlh-1 F20 (CD0134c)

(TGTTACACGAAAAATAGAT) n TGTTACACGAAAAATAGAT (TGTTACACGAAAAATAGAT) 2

1 1 1

D

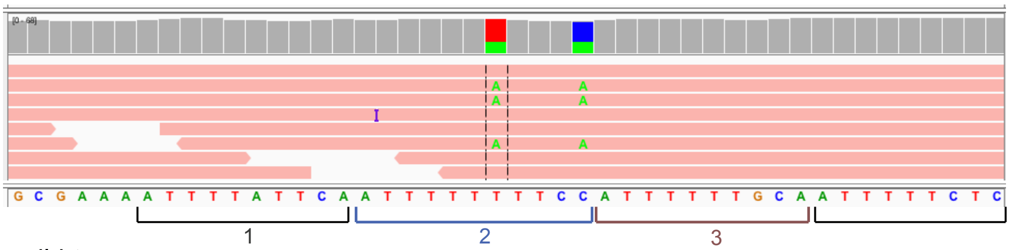

wild-type

ATTTTATTCA ATTTTTTTTCC ATTTTTTGCA

1 2 3

mlh-1 F20 (CD0134d)

ATTTTATTCA ATTTTTATTCA ATTTTTTGCA

1 1’ 3
