# Supplementary material for: Mutational signatures of DNA mismatch repair deficiency in C. elegans and human cancers
Source: Genome Res. 2018 May;28(5):666–75. doi: 10.1101/gr.226845.117 (PMC5932607; doi:10.1101/gr.226845.117)

Supplemental_Fig_S3

A

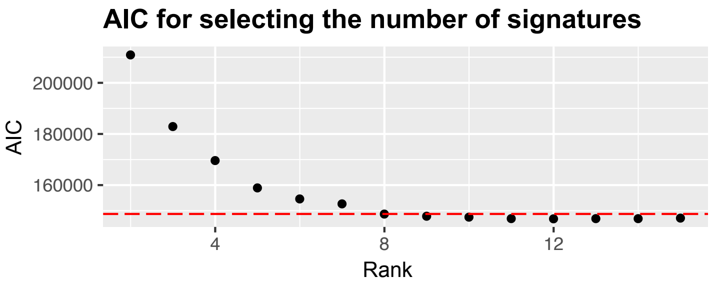

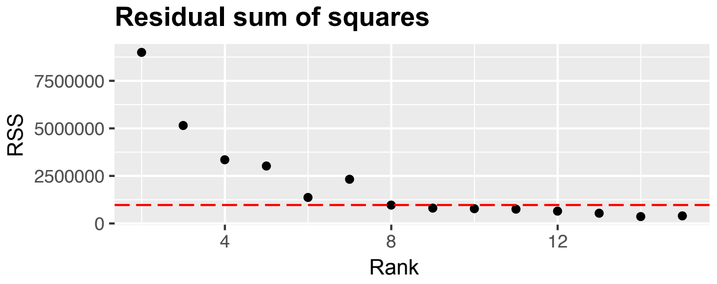

B

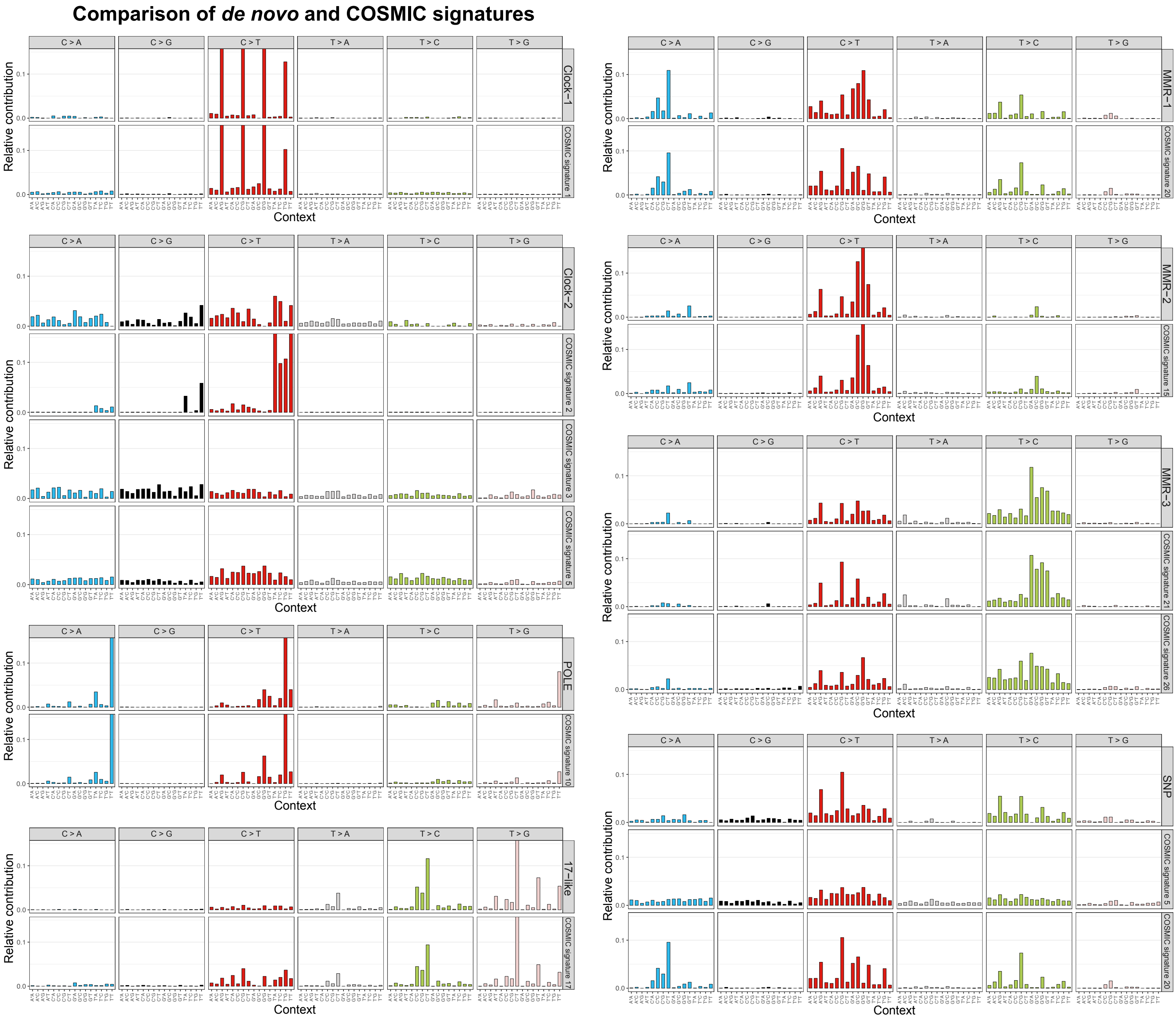

Supplement: Supplemental Material [file supp_gr.226845.117_Supplemental_Fig_S3.pdf]
