# Supplementary material for: Mutational signatures of DNA mismatch repair deficiency in C. elegans and human cancers
Source: Genome Res. 2018 May;28(5):666–75. doi: 10.1101/gr.226845.117 (PMC5932607; doi:10.1101/gr.226845.117)
Supplement: Supplemental Material [file supp_gr.226845.117_Supplemental_data_analysis.docx]

STAD and COAD analysis

N. Volkova, B. Meier, M. Gerstung

Apr 2017

Table of Contents

Get the data, signatures and contributions 1

Functions for plotting: 2

Functions for NMF: extracting signatures and calculating contributions. 15

Other 17

*C. elegans* MMR mutational patterns 20

ICGC data 21

Signature extraction 23

MMR 26

Amount of indels 28

More about signature contributions 32

Similarities 32

Age correlation 33

Average profiles 33

Cosine similarity simulations for signature comparison 34

Distribution of angles in COSMIC signatures 34

SNP contamination signature 40

Homopolymer analysis in *C. elegans* 43

Analysis of mutational signatures in COAD and STAD cancer datasets from [ICGC](http://icgc.org) and *C. elegans* MMR mutants for publication **Mutational signatures of DNA mismatch repair deficiency in *C. elegans* and human cancers** ([Meier, Volkova et al. 2017](https://www.biorxiv.org/content/biorxiv/early/2017/06/13/149153.full.pdf)).

Full version of the codes and complementary datasets are available in the [Github repository](https://github.com/gerstung-lab/MMR). In order to reproduce the analysis, install the Github repository, follow the instruction on data downloading, set it as working directory and run the codes there.

## Get the data, signatures and contributions

Preparation:

library(tsne)
library(NMF)
library(devtools)
#devtools::install_github("mg14/mg14") # for plotting purposes
library(mg14)
library(ggplot2)
library(reshape2)
library(VariantAnnotation)
library(deconstructSigs)
library(Biostrings)
library(GenomicRanges)
library(dplyr)
library(gam)

### Functions for plotting:

**plot_decomposition** - function for plotting decompositions of mutational profiles over different signatures

Requires: ggplot2, reshape2 libraries.

Arguments:

- decomposition - matrix of fractions, samples x signatures
- mm - matrix of mutational counts of different types, samples x 96 (or 104) mutation types
- intnames - character vector of names of samples to plot (should be present in decomposition and mm’s rownames)
- col - vector of colours to use for signatures
- circle - TRUE or FALSE, make it a circular barplot (‘nautilus-plot’) or a normal one
- size - regulates the size of x-axis text (sample names)
- axis.size - regulates the size og legend text and y-axis text (signature names and numbers on y scale)

plot_decomposition <- function(decomposition, mm, intnames, col,circle=F,size=6,axis.size=10) {
 for (i in 1:nrow(decomposition))
 decomposition[i,] = decomposition[i,] / sum(decomposition[i,])
 new.cont.mat <- t(decomposition[intnames,])
 for (y in colnames(new.cont.mat)) {
 new.cont.mat[,y] = new.cont.mat[,y] * rowSums(mm)[y]
 }
 m.new.cont.mat <- melt(new.cont.mat)
 colnames(m.new.cont.mat) = c("Signature","Sample","Contribution")
 order(rowSums(mm),decreasing = F) -> sampleorder
 names(sampleorder) <- row.names(mm)[sampleorder]
 plot = ggplot(m.new.cont.mat, aes(x = factor(Sample,levels=names(sampleorder)),
 y = Contribution, fill = factor(Signature,levels=colnames(decomposition)), order = Sample)) +
 geom_bar(stat = "identity", colour = "black") +
 labs(x = "", y = "Absolute contribution \n (no. mutations)") +
 theme_bw() +
 scale_fill_manual(name="",values=col) +
 theme(panel.grid.minor.x = element_blank(), panel.grid.major.x = element_blank()) +
 theme(panel.grid.minor.y = element_blank(), panel.grid.major.y = element_blank()) +
 theme(axis.text.x = element_text(angle = 90, hjust = 1,size=size),
 axis.text.y = element_text(size=axis.size),
 legend.text=element_text(size=axis.size))
 if (circle) {
 new.cont.mat <- t(decomposition[intnames,])
 for (y in colnames(new.cont.mat)) {
 new.cont.mat[,y] = new.cont.mat[,y] * log10(rowSums(mm)[y])
 }
 m.new.cont.mat <- melt(new.cont.mat)
 colnames(m.new.cont.mat) = c("Signature","Sample","Contribution")
 plot = ggplot(m.new.cont.mat, aes(x = factor(Sample,levels=names(sampleorder)),
 y = Contribution, fill = factor(Signature,levels=colnames(decomposition)), order = Sample)) +
 geom_bar(stat = "identity", colour = "black") +
 theme_bw() +
 scale_fill_manual(name="",values=col) +
 theme(panel.grid.minor.x = element_blank(), panel.grid.major.x = element_blank()) +
 theme(panel.grid.minor.y = element_blank(), panel.grid.major.y = element_blank()) +
 theme(axis.text.x = element_text(size=size),
 axis.text.y = element_text(size=axis.size),
 legend.text=element_text(size=axis.size)) +
 coord_polar() +
 labs(x = "", y = "Absolute contribution \n (no. mutations, log10)")
 }
 plot
}

**plot_sig_wb** - function for plotting 96-long signatures of relative contributions of different mutation types.

Requires: ggplot2 and reshape2 libraries.

Arguments:

- mut_matrix - matrix of signatures (or profiles), samples x signatures
- colors - colors to use for 6 mutation classes
- ymin, ymax - max and min value on the plot
- flip - FALSE or TRUE, flips the plot vertically
- size - size of mutation class titles

plot_sig_wb <- function (mut_matrix, colors, ymin=0,ymax = 0.15,flip=F,size=12) # plotting 96-signatures with no lines
{
 C_TRIPLETS = c(
 "ACA", "ACC", "ACG", "ACT",
 "CCA", "CCC", "CCG", "CCT",
 "GCA", "GCC", "GCG", "GCT",
 "TCA", "TCC", "TCG", "TCT")

 T_TRIPLETS = c(
 "ATA", "ATC", "ATG", "ATT",
 "CTA", "CTC", "CTG", "CTT",
 "GTA", "GTC", "GTG", "GTT",
 "TTA", "TTC", "TTG", "TTT")

 IND_TRIPLETS = c(
 "A*A", "A*C", "A*G", "A*T",
 "C*A", "C*C", "C*G", "C*T",
 "G*A", "G*C", "G*G", "G*T",
 "T*A", "T*C", "T*G", "T*T")

 TRIPLETS_96 = c(rep(C_TRIPLETS, 3), rep(T_TRIPLETS, 3))
 TRIPLETS_112 = c(rep(C_TRIPLETS, 3), rep(T_TRIPLETS, 3),IND_TRIPLETS)
 norm_mut_matrix = apply(mut_matrix, 2, function(x) x/sum(x))
 if (missing(colors)) {
 colors = c("#2EBAED", "#000000", "#DE1C14",
 "#D4D2D2", "#ADCC54", "#F0D0CE")
 }
 context = TRIPLETS_96
 substitution = rep(c("C>A","C>G","C>T","T>A","T>C","T>G"), each = 16)
 substring(context, 2, 2) = "*"
 df = data.frame(substitution = substitution, context = context)
 rownames(norm_mut_matrix) = NULL
 if (flip==T) norm_mut_matrix = -norm_mut_matrix
 df2 = cbind(df, as.data.frame(norm_mut_matrix))
 df3 = melt(df2, id.vars = c("substitution", "context"))
 value = NULL
 if (ymax>0) breaks = c(0,0.1) else breaks = c(-0.1,0)
 if (ymax>0) lbls = c('0','0.1') else lbls = c('0.1','0')
 plot = ggplot(data = df3, aes(x = context, y = value, fill = substitution, width = 0.6)) +
 geom_bar(stat = "identity", colour = "black",size = 0.2) +
 scale_fill_manual(values = colors) + facet_grid(variable ~ substitution) +
 ylab("Relative contribution") + coord_cartesian(ylim = c(ymin,ymax)) +
 guides(fill = FALSE) + theme_bw() + scale_y_continuous(breaks = breaks, labels = lbls) +
 theme(text = element_text(family='ArialMT'),
 axis.title.y = element_text(size = 14,vjust = 1),
 axis.text.y = element_text(size = 8),
 axis.title.x = element_text(size = 14),
 axis.text.x = element_text(size = 8, angle = 90, vjust = 0.4),
 strip.text.x = element_text(size = 16),
 strip.text.y = element_text(size = size),
 panel.grid.major.x = element_blank(),
 strip.background = element_blank(),
 panel.border = element_rect(colour="white"),
 panel.spacing = unit(0.1,'lines'))
 return(plot)
}

**plot_profiles** - function for plotting 96-long mutational profiles (in absolute numbers).

Requires: ggplot2 and reshape2 libraries.

Arguments:

- mut_matrix - matrix of signatures (or profiles), samples x signatures
- colors - colors to use for 6 mutation classes

plot_profiles <- function (mut_matrix, colors) # plot mutational profiles without normalizing
{
 C_TRIPLETS = c(
 "ACA", "ACC", "ACG", "ACT",
 "CCA", "CCC", "CCG", "CCT",
 "GCA", "GCC", "GCG", "GCT",
 "TCA", "TCC", "TCG", "TCT")

 T_TRIPLETS = c(
 "ATA", "ATC", "ATG", "ATT",
 "CTA", "CTC", "CTG", "CTT",
 "GTA", "GTC", "GTG", "GTT",
 "TTA", "TTC", "TTG", "TTT")

 TRIPLETS_96 = c(rep(C_TRIPLETS, 3), rep(T_TRIPLETS, 3))
 norm_mut_matrix = apply(mut_matrix, 2, function(x) x/sum(x))
 if (missing(colors)) {
 colors = c(
 "#2EBAED", "#000000", "#DE1C14",
 "#D4D2D2", "#ADCC54", "#F0D0CE")
 }
 if (length(colors) != 6) {
 stop("Provide colors vector with length 6")
 }
 context = TRIPLETS_96
 substitution = rep(c("C>A","C>G","C>T","T>A","T>C","T>G"), each = 16)
 substring(context, 2, 2) = "*"
 df = data.frame(substitution = substitution, context = context)
 df2 = cbind(df, as.data.frame(mut_matrix))
 df3 = melt(df2, id.vars = c("substitution", "context"))
 value = NULL
 plot = ggplot(data = df3, aes(x = context, y = value, fill = substitution, width = 0.6)) +
 geom_bar(stat = "identity", colour = "black",size = 0.2) +
 scale_fill_manual(values = colors) + facet_grid(variable ~ substitution) +
 ylab("Relative contribution") + coord_cartesian(ylim = c(0,max(mut_matrix))) +
 guides(fill = FALSE) + theme_bw() +
 theme(axis.title.y = element_text(size = 14,vjust = 1), axis.text.y = element_text(size = 10), axis.title.x = element_text(size = 14),
 axis.text.x = element_text(size = 7, angle = 90, vjust = 0.4),
 strip.text.x = element_text(size = 11), strip.text.y = element_text(size = 13),
 panel.grid.major.x = element_blank())
 return(plot)
}

**plot_sig_104** - function for plotting 104-long signatures of relative contributions of different mutation types.

Requires: ggplot2 and reshape2 libraries.

Arguments:

- mut_matrix - matrix of signatures (or profiles), mutation types x signatures
- colors - colors to use for 8 mutation classes
- size - size of mutation class titles
- ymax - maximal value on the plot

plot_sig_104 <- function(mut_matrix,colors=NA,size=8,ymax=0.2) { # plot 104-signatures

 C_TRIPLETS = c(
 "ACA", "ACC", "ACG", "ACT",
 "CCA", "CCC", "CCG", "CCT",
 "GCA", "GCC", "GCG", "GCT",
 "TCA", "TCC", "TCG", "TCT")

 T_TRIPLETS = c(
 "ATA", "ATC", "ATG", "ATT",
 "CTA", "CTC", "CTG", "CTT",
 "GTA", "GTC", "GTG", "GTT",
 "TTA", "TTC", "TTG", "TTT")

 if (is.na(colors)) colors=c("#2EBAED", "#000000", "#DE1C14","orange","purple","#D4D2D2", "#ADCC54", "#F0D0CE")

 types <- c(rep(C_TRIPLETS,3), rep(T_TRIPLETS,3))
 norm_mut_matrix = apply(mut_matrix, 2, function(x) x/sum(x))
 df = as.data.frame(norm_mut_matrix)
 df$Type = c(rep(c("C>A","C>G","C>T","T>A","T>C","T>G"),each=16),rep("INS",4),rep("DEL",4))
 df$Base = c(types,rep(c("A","C","G","T"),2))
 substring(df$Base[nchar(df$Base)>1], 2, 2) = "*"
 #colnames(df)[1:ncol(mut_matrix)] = paste0("Signature.",c(1:ncol(mut_matrix)),sep="")
 df2 = melt(df,id.vars = c("Type","Base"))
 df2$Type_f = factor(df2$Type, levels=c("C>A","C>G","C>T","T>A","T>C","T>G","DEL","INS"))
 plot = ggplot(data = df2, aes(x = Base, y = value, fill = Type, width = 0.6)) +
 geom_bar(stat = "identity", colour = "black",size = 0.2) +
 scale_fill_manual(values = colors) +
 facet_grid(variable ~ Type_f,scales = "free_x") +
 ylab("Relative contribution") + coord_cartesian(ylim = c(0,ymax)) +
 guides(fill = FALSE) + theme_bw() + scale_y_continuous(breaks = c(0,0.15)) +
 theme(text = element_text(family='ArialMT'),
 axis.title.y = element_text(size = 14,vjust = 1),
 axis.text.y = element_text(size = 8),
 axis.title.x = element_text(size = 14),
 axis.text.x = element_text(size = 8, angle = 90, vjust = 0.4),
 strip.text.x = element_text(size = 16),
 strip.text.y = element_text(size = size),
 panel.grid.major.x = element_blank(),
 strip.background = element_blank(),
 panel.border = element_rect(colour="white"),
 panel.spacing = unit(0.1,'lines'))
 return(plot)
}

**plot_sig_104_CI** - function for plotting 104-long signatures of relative contributions of different mutation types with confidence intervals.

Requires: ggplot2 and reshape2 libraries.

Arguments:

- mut_matrix - matrix of signatures (or profiles), mutation types x signatures
- mut_matrix_lower - matrix of lower CI values for signatures, mutation types x signatures
- mut_matrix_upper - matrix of upper CI values for signatures, mutation types x signatures
- colors - colors to use for 8 mutation classes
- size - size of mutation class titles
- ymax - maximal value on the plot

plot_sig_104_CI <- function(mut_matrix,mut_matrix_lower,mut_matrix_upper,size=8,ymax=0.2,colors) { # plot 104-signatures with confidence intervals

 colors = c("#2EBAED", "#000000", "#DE1C14","orange","purple","#D4D2D2", "#ADCC54", "#F0D0CE")


 C_TRIPLETS = c(
 "ACA", "ACC", "ACG", "ACT",
 "CCA", "CCC", "CCG", "CCT",
 "GCA", "GCC", "GCG", "GCT",
 "TCA", "TCC", "TCG", "TCT")

 T_TRIPLETS = c(
 "ATA", "ATC", "ATG", "ATT",
 "CTA", "CTC", "CTG", "CTT",
 "GTA", "GTC", "GTG", "GTT",
 "TTA", "TTC", "TTG", "TTT")

 types <- c(rep(C_TRIPLETS,3), rep(T_TRIPLETS,3))
 df = as.data.frame(mut_matrix)
 df$Type = c(rep(c("C>A","C>G","C>T","T>A","T>C","T>G"),each=16),rep("INS",4),rep("DEL",4))
 df$Base = c(types,rep(c("A","C","G","T"),2))
 substring(df$Base[nchar(df$Base)>1], 2, 2) = "*"
 df2 = melt(df,id.vars = c("Type","Base"))
 df2$Type_f = factor(df2$Type, levels=c("C>A","C>G","C>T","T>A","T>C","T>G","DEL","INS"))
 plot = ggplot(data = df2, aes(x = Base, y = value, fill = Type, width = 0.6)) +
 geom_bar(stat = "identity", colour = "black",size = 0.2) +
 scale_fill_manual(values = colors) +
 facet_grid(variable ~ Type_f,scales = "free_x") +
 ylab("Relative contribution") + coord_cartesian(ylim = c(0,ymax)) +
 guides(fill = FALSE) + theme_bw() +
 theme(axis.title.y = element_text(size = 14,vjust = 1), axis.text.y = element_text(size = 6), axis.title.x = element_text(size = 14),
 axis.text.x = element_text(size = 6, angle = 90, vjust = 0.4),
 strip.text.x = element_text(size = 10), strip.text.y = element_text(size = size),
 panel.grid.major.x = element_blank())

 mut_matrix_lower <- mut_matrix_lower[row.names(mut_matrix),]
 mut_matrix_upper <- mut_matrix_upper[row.names(mut_matrix),]
 rownames(mut_matrix_lower) = rownames(mut_matrix_upper) = NULL
 df_lower = as.data.frame(mut_matrix_lower)
 df_upper = as.data.frame(mut_matrix_upper)
 df_lower$Type = c(rep(c("C>A","C>G","C>T","T>A","T>C","T>G"),each=16),rep("INS",4),rep("DEL",4))
 df_lower$Base = c(types,rep(c("A","C","G","T"),2))
 df_upper$Type = c(rep(c("C>A","C>G","C>T","T>A","T>C","T>G"),each=16),rep("INS",4),rep("DEL",4))
 df_upper$Base = c(types,rep(c("A","C","G","T"),2))
 df_lower = melt(df_lower, id.vars = c("Type","Base"))
 df_upper = melt(df_upper, id.vars = c("Type","Base"))
 df3 <- cbind(df2, value_min = df_lower$value, value_max = df_upper$value)
 df3$Type_f = factor(df3$Type, levels=c("C>A","C>G","C>T","T>A","T>C","T>G","DEL","INS"))
 plot2 = plot + geom_pointrange(data=df3, aes(ymin=value,ymax=value_max,colour=factor(Type)), fatten = 0.01, size=0.5, show.legend = F, colour="white") +
 scale_color_manual(values=colors) +
 geom_pointrange(data=df3, aes(ymin=value_min,ymax=value), size=0.5, fatten = 0.01,show.legend = F, colour="white")

 return(plot2)
}

**plot_profiles_104** - function for plotting 104-long mutational profiles (normalized, i.e. in relative contribution).

Requires: ggplot2 and reshape2 libraries.

Arguments:

- mut_matrix - matrix of profiles, samples x mutation counts
- colors - colors to use for 8 mutation classes
- boxplot - FALSE or TRUE, merges all the values across profiles into a boxplot
- normalize - FALSE or TRUE, turns absolute numbers into relative contributions
- size - regulates font size of the text on the plot

plot_profiles_104 <- function (mut_matrix, colors=NA, boxplot=F, normalize=F, size=6) # plot 104-long mutational profiles
{

 if (is.na(colors)) colors = c("#2EBAED", "#000000", "#DE1C14","orange","purple","#D4D2D2", "#ADCC54", "#F0D0CE")

 C_TRIPLETS = c(
 "ACA", "ACC", "ACG", "ACT",
 "CCA", "CCC", "CCG", "CCT",
 "GCA", "GCC", "GCG", "GCT",
 "TCA", "TCC", "TCG", "TCT")

 T_TRIPLETS = c(
 "ATA", "ATC", "ATG", "ATT",
 "CTA", "CTC", "CTG", "CTT",
 "GTA", "GTC", "GTG", "GTT",
 "TTA", "TTC", "TTG", "TTT")

 types <- c(rep(C_TRIPLETS,3), rep(T_TRIPLETS,3))
 if (normalize) {
 mut_matrix <- apply(mut_matrix, 2, function(x) x/sum(x))
 }
 df = as.data.frame(mut_matrix)
 df$Type = c(rep(c("C>A","C>G","C>T","T>A","T>C","T>G"),each=16),rep("INS",4),rep("DEL",4))
 df$Base = c(types,rep(c("A","C","G","T"),2))
 substring(df$Base[nchar(df$Base)>1], 2, 2) = "*"
 df2 = melt(df,id.vars = c("Type","Base"))
 df2$Type_f = factor(df2$Type, levels=c("C>A","C>G","C>T","T>A","T>C","T>G","DEL","INS"))
 if (boxplot) {
 plot = ggplot(data = df2, aes(x = Base, y = value, fill=Type)) +
 geom_boxplot() +
 scale_fill_manual(values = colors) +
 facet_grid(. ~ Type_f, scales="free") +
 ylab("Mutation counts") +
 guides(fill = FALSE) + theme_bw() +
 theme(axis.title.y = element_text(size = 20,vjust = 1), axis.text.y = element_text(size = 16), axis.title.x = element_text(size = 20),
 axis.text.x = element_text(size = 8, angle = 90, vjust = 0.4),
 strip.text.x = element_text(size = size), strip.text.y = element_text(size = size),
 panel.grid.major.x = element_blank())
 } else {
 plot = ggplot(data = df2, aes(x = Base, y = value, fill = Type, width = 0.6)) +
 geom_bar(stat = "identity", colour = "black",size = 0.2) +
 scale_fill_manual(values = c("#2EBAED", "#000000", "#DE1C14","orange","purple","#D4D2D2", "#ADCC54", "#F0D0CE")) +
 facet_grid(variable ~ Type_f,scales = "free_x") +
 ylab("Mutation counts") + coord_cartesian(ylim = c(0,max(mut_matrix))) +
 guides(fill = FALSE) + theme_bw() +
 theme(axis.title.y = element_text(size = 14,vjust = 1), axis.text.y = element_text(size = 6), axis.title.x = element_text(size = 14),
 axis.text.x = element_text(size = 6, angle = 90, vjust = 0.4),
 strip.text.x = element_text(size = 6), strip.text.y = element_text(size = 6),
 panel.grid.major.x = element_blank())
 }
 return(plot)
}

**plot_104_profile_CI** - function for plotting 104-long mutational profiles (in absolute numbers) with confidence intervals.

Requires: ggplot2 and reshape2 libraries.

Arguments:

- mut_matrix - matrix of profiles, samples x mutation counts
- colors - colors to use for 8 mutation classes

plot_104_profile_CI <- function (mut_matrix, mut_matrix_lower = NULL,
 mut_matrix_upper = NULL,
 colors=c("deepskyblue2","black","red3","orange","purple","grey","olivedrab3","pink"),size=6,ymax=0.25) # plot 104-long profiles with confidence intervals
{
 C_TRIPLETS = c(
 "ACA", "ACC", "ACG", "ACT",
 "CCA", "CCC", "CCG", "CCT",
 "GCA", "GCC", "GCG", "GCT",
 "TCA", "TCC", "TCG", "TCT")

 T_TRIPLETS = c(
 "ATA", "ATC", "ATG", "ATT",
 "CTA", "CTC", "CTG", "CTT",
 "GTA", "GTC", "GTG", "GTT",
 "TTA", "TTC", "TTG", "TTT")


 norm_mut_matrix = mut_matrix
 mult <- colSums(norm_mut_matrix)
 norm_mut_matrix = apply(mut_matrix, 2, function(x) x/sum(x))
 types <- c(rep(C_TRIPLETS,3), rep(T_TRIPLETS,3))
 df = as.data.frame(norm_mut_matrix)
 df$Type = c(rep(c("C>A","C>G","C>T","T>A","T>C","T>G"),each=16),rep("INS",4),rep("DEL",4))
 df$Base = c(types,rep(c("A","C","G","T"),2))
 df2 = melt(df,id.vars = c("Type","Base"))
 df2$Type_f = factor(df2$Type, levels=c("C>A","C>G","C>T","T>A","T>C","T>G","DEL","INS"))
 plot = ggplot(data = df2, aes(x = Base, y = value, fill = Type, width = 0.6)) +
 geom_bar(stat = "identity", colour = "black",size = 0.2) +
 scale_fill_manual(values = colors) +
 facet_grid(variable ~ Type_f,scales = "free_x") +
 ylab("Mutation counts") + coord_cartesian(ylim = c(0,ymax)) +
 guides(fill = FALSE) + theme_bw() +
 theme(axis.title.y = element_text(size = 16,vjust = 1), axis.text.y = element_text(size = size), axis.title.x = element_text(size = 16),
 axis.text.x = element_text(size = size, angle = 90, vjust = 0.4),
 strip.text.x = element_text(size = size), strip.text.y = element_text(size = size),
 panel.grid.major.x = element_blank())

 mut_matrix_lower <- mut_matrix_lower[row.names(mut_matrix),]
 mut_matrix_upper <- mut_matrix_upper[row.names(mut_matrix),]
 norm_mut_matrix_lower <- mut_matrix_lower
 norm_mut_matrix_upper <- mut_matrix_upper
 for (i in 1:ncol(norm_mut_matrix_lower)) {
 norm_mut_matrix_lower[,i] = norm_mut_matrix_lower[,i] / mult[colnames(norm_mut_matrix_lower)[i]]
 norm_mut_matrix_upper[,i] = norm_mut_matrix_upper[,i] / mult[colnames(norm_mut_matrix_lower)[i]]
 }
 rownames(norm_mut_matrix_upper) = rownames(norm_mut_matrix_lower) = NULL
 df_lower = as.data.frame(norm_mut_matrix_lower)
 df_upper = as.data.frame(norm_mut_matrix_upper)
 df_lower$Type = c(rep(c("C>A","C>G","C>T","T>A","T>C","T>G"),each=16),rep("INS",4),rep("DEL",4))
 df_lower$Base = c(types,rep(c("A","C","G","T"),2))
 df_upper$Type = c(rep(c("C>A","C>G","C>T","T>A","T>C","T>G"),each=16),rep("INS",4),rep("DEL",4))
 df_upper$Base = c(types,rep(c("A","C","G","T"),2))
 df_lower = melt(df_lower, id.vars = c("Type","Base"))
 df_upper = melt(df_upper, id.vars = c("Type","Base"))
 df3 <- cbind(df2, value_min = df_lower$value, value_max = df_upper$value)
 plot2 = plot + geom_pointrange(data=df3, aes(ymin=value,ymax=value_max,colour = Type_f), fatten = 0.01, size=1, show.legend = F) +
 scale_color_manual(values=c(colors,"white")) +
 geom_pointrange(data=df3, aes(ymin=value_min,ymax=value,colour="white"), size=1, fatten = 0.01,show.legend = F)

 return(plot2)
}

**plot_96_profile_CI** - function for plotting 96-long mutational profiles of different mutation types with confidence intervals.

Requires: ggplot2 and reshape2 libraries.

Arguments:

- mut_matrix - matrix of profiles, samples x mutation counts
- mut_matrix_lower - matrix of lower CI values for signatures, mutation types x samples
- mut_matrix_upper - matrix of upper CI values for signatures, mutation types x samples
- colors - colors to use for 6 mutation classes
- size - size of mutation class titles
- ymax - maximal value on the plot
- CI - FALSE or TRUE, do not / do plot CIs

plot_96_profile_CI <- function (mut_matrix, mut_matrix_lower = NULL,
 mut_matrix_upper = NULL, CI=FALSE,
 colors=c("deepskyblue2","black","red3","grey","olivedrab3","pink"), ymax=NA, size=9)
{
 if(is.na(ymax)) ymax = max(mut_matrix_upper)
 if (ymax>1) step = 10
 if (ymax<=1) step = 0.1
 C_TRIPLETS = c(
 "ACA", "ACC", "ACG", "ACT",
 "CCA", "CCC", "CCG", "CCT",
 "GCA", "GCC", "GCG", "GCT",
 "TCA", "TCC", "TCG", "TCT")
 T_TRIPLETS = c(
 "ATA", "ATC", "ATG", "ATT",
 "CTA", "CTC", "CTG", "CTT",
 "GTA", "GTC", "GTG", "GTT",
 "TTA", "TTC", "TTG", "TTT")
 types <- c(rep(C_TRIPLETS,3), rep(T_TRIPLETS,3))
 context = types
 substitution = rep(c("C>A","C>G","C>T","T>A","T>C","T>G"), each = 16)
 substring(context, 2, 2) = "*"
 df = data.frame(substitution = substitution, context = context)
 df2 = cbind(df, as.data.frame(mut_matrix))
 df3 = melt(df2, id.vars = c("substitution", "context"))
 value = NULL
 plot = ggplot(data = df3, aes(x = context, y = value, fill = substitution, width = 0.6)) +
 geom_bar(stat = "identity", colour = "black", size = 0.2) +
 scale_fill_manual(values = colors) +
 facet_grid(variable ~ substitution) +
 coord_cartesian(ylim = c(0,ymax)) +
 scale_y_continuous(breaks = seq(0, ymax, step)) +
 guides(fill = FALSE) + theme_bw() +
 theme(text = element_text(family='ArialMT'),
 axis.title.y = element_text(size = 14,vjust = 1),
 axis.text.y = element_text(size = 8),
 axis.title.x = element_text(size = 14),
 axis.text.x = element_text(size = 8, angle = 90, vjust = 0.4),
 strip.text.x = element_text(size = 16),
 strip.text.y = element_text(size = size),
 panel.grid.major.x = element_blank(),
 strip.background = element_blank(),
 panel.border = element_rect(colour="white"),
 panel.spacing = unit(0.1,'lines')) +
 labs(y="Number of mutations", x="Context")

 if (CI) { # add confidence intervals
 rownames(mut_matrix_upper) = rownames(mut_matrix_lower) = NULL
 df_lower = cbind(df, as.data.frame(mut_matrix_lower))
 df_upper = cbind(df, as.data.frame(mut_matrix_upper))
 df_lower = melt(df_lower, id.vars = c("substitution", "context"))
 df_upper = melt(df_upper, id.vars = c("substitution", "context"))
 df2 = cbind(df, as.data.frame(mut_matrix[,colnames(mut_matrix_lower)]))
 df3 = melt(df2, id.vars = c("substitution", "context"))
 df3 <- cbind(df3, value_min = df_lower$value, value_max = df_upper$value)
 plot = plot + geom_pointrange(data=df3, aes(ymin=value,ymax=value_max,colour = substitution), size=1.5, fatten = 0.001,show.legend = F) +
 scale_color_manual(values=c(colors,"white")) +
 geom_pointrange(data=df3, aes(ymin=value_min,ymax=value,colour="white"), size=1.5, fatten = 0.001,show.legend = F)
 }
 return(plot)
}

### Functions for NMF: extracting signatures and calculating contributions.

**nmSolve** - extraction of signatures for given exposures.

Arguments:

- D - matrix of mutation counts, samples x mutation types
- P - exposures, samples x signatures
- maxIter - maximal number of iterations in NMF algorithm
- tol - lower bound for the change of signature matrix between iterations
- div.err - lower bound for the change of the divergence between D and its factorization between iterations

nmSolve <- function(D, P, maxIter = 10000, tol=1e-5, div.err=1e-7) {
 n <- nrow(D)
 mask <- !is.na(D)
 m <- ncol(D)
 s <- ncol(P)
 rP <- rep(colSums(P), m)
 tP <- t(P)
 D <- as.matrix(D)
 P <- as.matrix(P)
 E1 <- E2 <- matrix(runif(s * m, 1e-3, 1), ncol = m)
 err <- 2*tol
 D[is.na(D)] <- 0

 iter <- 1
 divergence.old <- mean(D*log(D/(P %*% (E2 + .Machine$double.eps))) - D + P%*%E2, na.rm=T)
 div.change <- 2 * div.err

 while (iter < maxIter & err > tol & abs(div.change) > div.err) {
 E1 <- E2
 E2 <- E1 * (tP %*% ((mask*D)/(mask*(P %*% (E1 + .Machine$double.eps)) + .Machine$double.eps)))/rP
 iter <- iter + 1
 err <- mean(abs(E2 - E1)/(E1+.Machine$double.eps), na.rm=TRUE)
 divergence <- mean(D*log(D/(P %*% (E2 + .Machine$double.eps))) - D + P%*%E2, na.rm=T) # KL distance from D to P%*%E2
 div.change <- divergence.old - divergence
 divergence.old = divergence
 if(iter %% 100 == 0) cat(round(-log10(err)))
 }
 cat("\n")
 if(iter == maxIter) warning(paste("No convergence after",iter, "iterations."))
 E2
}

**nmFit** - fitting the signatures (extracting exposures).

Arguments:

- D - matrix of mutation counts, samples x mutation types
- E - signatures, signatures x mutation types
- maxIter - maximal number of iterations in NMF algorithm
- tol - lower bound for the change of exposure matrix between iterations
- div.err - lower bound for the change of the divergence between D and its factorization between iterations

nmFit <- function(D, E, maxIter = 10000, tol=1e-5, div.err=1e-7) {
 n <- nrow(D)
 m <- ncol(D)
 s <- nrow(E)
 tE <- t(E)
 rE <- rep(rowSums(E),each=n)
 D <- as.matrix(D)
 E <- as.matrix(E)
 P1 <- P2 <- matrix(runif(s * n, 1e-3, 1), nrow = n)
 err <- 2*tol

 iter <- 1
 divergence.old <- mean(D * log(D/(P2%*%(E + .Machine$double.eps))) - D + P2%*%E, na.rm=T)
 div.change <- 2 * div.err
 while (iter < maxIter & err > tol & abs(div.change) > div.err) {
 P1 <- P2
 P2 <- P1 * ((D/((P1 +.Machine$double.eps) %*% (E +.Machine$double.eps))) %*% tE) / (rE+.Machine$double.eps)
 iter <- iter + 1
 err <- mean(abs(P2 - P1)/(P1+.Machine$double.eps), na.rm=TRUE)
 divergence <- mean(D*log(D/((P2 + .Machine$double.eps)%*%(E+.Machine$double.eps))) - D + P2%*%E, na.rm=T) # KL distance from D to P%*%E
 div.change <- divergence.old - divergence
 divergence.old = divergence
 if(iter %% 100 == 0) cat(round(-log10(err)))
 # add likelihood convergence
 }
 cat("\n")
 if(iter == maxIter) warning(paste("No convergence after",iter, "iterations."))
 P2
}

### Other

Technical function: slightly modified function from mg14 to plot cirles with different outline colour.

corr_scatterpie <- function (x, y, p, r, xlab = "", ylab = "", circles = FALSE,
 lwd.circle = rep(1, length(x)), lty.circle = rep(1, length(x)),
 add = FALSE, col.circle='black', ...)
{
 if (!add)
 plot(x, y, xlab = xlab, ylab = ylab, pch = NA)
 for (i in seq_along(x)) {
 mg14:::.pie(p[i, ], x0 = x[i], y0 = y[i], radius = r[i], add = TRUE,
 ...)
 if (circles) {
 u <- par("usr")
 pr <- (u[2] - u[1])/(u[4] - u[3])
 fr <- par("pin")[1]/par("pin")[2]
 polygon(x[i] + cos(seq(0, 2 * pi, l = 100)) * r[i],
 y[i] + sin(seq(0, 2 * pi, l = 100)) * r[i]/pr *
 fr, col = NA, lty = lty.circle[i], lwd = lwd.circle[i], border=col.circle)
 }
 }
}

Get COSMIC signatures:

sp_url <- paste("http://cancer.sanger.ac.uk/cancergenome/assets/",
 "signatures_probabilities.txt", sep = "")
cancer_signatures = read.table(sp_url, sep = "\t", header = TRUE)
cancer_signatures = cancer_signatures[order(cancer_signatures[,1]),]
types <- as.character(cancer_signatures$Trinucleotide) # trinucleotide classes
types.full <- as.character(cancer_signatures$Somatic.Mutation.Type) # substitution types
row.names(cancer_signatures) <- types.full
cancer_signatures = as.matrix(cancer_signatures[,4:33])

Adjust them for exome trinucleotide frequencies:

cancer_signatures_adj <- cancer_signatures
for (i in 1:ncol(cancer_signatures)) {
 cancer_signatures_adj[,i] <- cancer_signatures_adj[,i] / tri.counts.genome[types,1] * tri.counts.exome[types,1]
 cancer_signatures_adj[,i] <- cancer_signatures_adj[,i] / sum(cancer_signatures_adj[,i])
}

Prepare human exome counts (regions taken from [Agilent SureSelect V5 Human All Exon](https://earray.chem.agilent.com/suredesign/index.htm), human genome sequence - from hg19 build)

ref_genome="BSgenome.Hsapiens.UCSC.hg19"
library(BSgenome.Hsapiens.UCSC.hg19)
exome <- read.table("S04380110_Covered.bed",header = FALSE, sep="\t",stringsAsFactors=FALSE, skip=2,quote="")
a <- getSeq(get(ref_genome))
a <- a[1:23] # get rid of Y
gr <- as(seqinfo(a), "GRanges") # turn into GRanges object
genome(gr) <- "hg19"
# Get the sequence for well covered exome
exactexomelist <- list()
for (j in 1:23) {
 tmp <- exome[exome$V1==seqlevels(a)[j],]
 exactexomelist[[j]] <- lapply(1:nrow(tmp), function(i)
 a[[j]][tmp$V2[i]:tmp$V3[i]])
}

load('exome.RData')
# nucleotide counts
exome.bases <- rowSums(sapply(1:23, function(k) rowSums(sapply(exactexomelist[[k]],oligonucleotideFrequency,width=1))))
ref_genome="BSgenome.Hsapiens.UCSC.hg19"
library(BSgenome.Hsapiens.UCSC.hg19)
#exome <- read.table("S04380110_Covered.bed",header = FALSE, sep="\t",stringsAsFactors=FALSE, skip=2,quote="")
a <- getSeq(get(ref_genome))
a <- a[1:23] # get rid of Y
gr <- as(seqinfo(a), "GRanges") # turn into GRanges object
genome(gr) <- "hg19"

Prepare *C. elegans* counts to account for trinucleotide content difference:

WBcel235 <- readDNAStringSet("C. elegans data/Caenorhabditis_elegans.WBcel235.dna_sm.toplevel.fa.gz")
worm.trinucleotides <- colSums(trinucleotideFrequency(WBcel235)[-5,])
human.trinucleotides <- as.vector(t(tri.counts.genome))
names(human.trinucleotides) <- row.names(tri.counts.genome)
trinucleotide.freq.factor <- sapply(unique(types), function(x) {
 freq.worm <- worm.trinucleotides[x] + worm.trinucleotides[as.character(reverseComplement(DNAString(x)))]
 return(freq.worm / human.trinucleotides[x]) # tri.counts.genome is already classified w.r.t. pyrimidine reference
})
human.trinucleotides <- as.vector(t(tri.counts.exome)) # / sum(tri.counts.genome))) # counts from "deconstructSigs" package
names(human.trinucleotides) <- row.names(tri.counts.exome)
trinucleotide.freq.factor.ex <- sapply(unique(types), function(x) {
 freq.worm <- worm.trinucleotides[x] + worm.trinucleotides[as.character(reverseComplement(DNAString(x)))]
 return(freq.worm / human.trinucleotides[x]) # tri.counts.genome is already classified w.r.t. pyrimidine reference
})
names(trinucleotide.freq.factor.ex) = names(trinucleotide.freq.factor) <- unique(types)

Visualize trinucleotide differences between *C. elegans* genome and human exome:

worm.trinucleotides.32 <- sapply(unique(types), function(x) {
 return(worm.trinucleotides[x] + worm.trinucleotides[as.character(reverseComplement(DNAString(x)))])
})
names(worm.trinucleotides.32) <- unique(types)
types.order <- c(rep(types[1:16],3),rep(types[49:64],3))
# human.trinucleotides in deconstructSigs

df = data.frame(worm.counts=c(worm.trinucleotides.32[types.order]),
 human.counts=c(t(tri.counts.exome)[1,][types.order]),
 Type = rep(c('C>A', 'C>G', 'C>T', 'T>A', 'T>C', 'T>G'), each = 16),
 Trinucleotide = c(types.order))
df$worm.counts <- df$worm.counts / sum(df$worm.counts[33:64])
df$human.counts = df$human.counts / sum(df$human.counts[33:64])
df$human.counts = -df$human.counts

df <- rbind(df, df[,c(2,1,3,4)])
df[97:192,1] <- -df[97:192,1]
df <- df[,-2]
df$Species <- c(rep("C. elegans",96), rep("Homo sapiens exome",96))
colnames(df) <- c('Fraction', 'Type', 'Trinucleotide', 'Species')
rownames(df) = NULL

p <- ggplot(data = df,aes(x = Trinucleotide,y=Fraction,fill=Species)) +
 geom_bar(stat="identity",colour="black",position = "dodge",size=0.1,width = 0.5) +
 scale_fill_manual(values = c("darkred","lightblue")) +
 facet_grid(Species ~ Type, scales = "free") +
 scale_x_discrete(labels=types.order) +
 theme_bw() + coord_cartesian() +
 theme(text = element_text(family='ArialMT'),
 axis.text=element_text(size=12),
 axis.text.x = element_text(angle = 90, vjust = 0.4,size=6),
 strip.text = element_text(size = 12),
 axis.title=element_text(size=12,face="bold"),
 legend.text = element_text(size=12),
 legend.title = element_text(size=24,face="bold"),
 panel.grid = element_blank(),
 strip.background = element_rect(colour='white', fill='white'),
 panel.border = element_rect(colour='black', size=0.1),
 panel.spacing = unit(0.01,'lines')) +
 guides(fill=F)
p

## *C. elegans* MMR mutational patterns

Upload mutation counts from *C. elegans* samples and calculate *C. elegans* signatures using additive Poisson model:

$Y_{i,j}=Pois(\lambda_{i,j})$,

$E[Y_{i,j}]=N\cdot(\beta_{j,b}+X_{g_{1}}\beta_{j,g_{1}}+X_{g_{2}}\beta_{j,g_{2}}+X_{g_{1}:g_{2}}\beta_{j,g_{1}:g_{2}})$,

where $\beta_{j,\cdot}\geq0$ - effects, $N$ - generation number, $g_{1}$, $g_{2}$ - genetic backgrounds, $b$ - background contribution, $X_{...}\in0,1$ indicates the presence of particular factors.

load("C. elegans data/Learned_signatures.RData")
# Contains mutation counts matrix mut_mat and exposure matrix small.X for the samples from C. elegans MMR and pole-4;pms-2 experiments.
learned.sigs <- nmSolve(t(mut_mat),small.X,maxIter=10000, tol = 1e-06, div.err = 1e-10)
for (i in 1:nrow(learned.sigs)) {
 learned.sigs[i,] <- learned.sigs[i,] / sum(learned.sigs[i,])
}

plot_sig_wb(t(learned.sigs))

Humanize the signatures:

learned.sigs.exome <- learned.sigs
for (i in 1:nrow(learned.sigs.exome)) {
 learned.sigs.exome[i,] <- learned.sigs.exome[i,] / trinucleotide.freq.factor.ex[types]
}
plot_sig_wb(t(learned.sigs.exome))

## ICGC data

ICGC data for COAD and STAD dataset were downloaded from [ICGC DCC](http://dcc.icgc.org) using COAD-US and STAD-US projects with WXS analysis type, respectively. The vcf file describing all somatic mutations across ICGC dataset is stored [here](https://dcc.icgc.org/releases/current/Summary) under the ‘simple_somatic_mutation.aggregated.vcf.gz’ name.

# download and read huge aggregated variant file
big.vcf <- readVcf("simple_somatic_mutation.aggregated.vcf")
mutations.COAD <- read.table(file="COAD/simple_somatic_mutation.open.tsv",sep="\t",header=T)
mutations.STAD <- read.table(file="STAD/simple_somatic_mutation.open.tsv",sep="\t",header=T)
per_sample_list <- sapply(unique(mutations.COAD$icgc_donor_id), function(donor) {
 unique(mutations.COAD$icgc_mutation_id[mutations.COAD$icgc_donor_id==donor])
})
names(per_sample_list) <- unique(mutations.COAD$icgc_donor_id)
vcf_list_COAD <- sapply(per_sample_list, function(x) big.vcf[as.character(x)])
names(vcf_list_COAD) <- names(per_sample_list)
vcf_list_COAD <- sapply(vcf_list_COAD, rowRanges)
for (sample in names(vcf_list_COAD)) {
 seqlevels(vcf_list_COAD[[sample]]) <- seqnames(get(ref_genome))[c(as.numeric(seqlevels(vcf_list_COAD[[sample]])[1:22]),25,23,24)]
 vcf_list_COAD[[sample]] <- vcf_list_COAD[[sample]][seqnames(vcf_list_COAD[[sample]])!="chrM"]
 vcf_list_COAD[[sample]] <- vcf_list_COAD[[sample]][seqnames(vcf_list_COAD[[sample]])!="chrY"]
}
per_sample_list <- sapply(unique(mutations.STAD$icgc_donor_id), function(donor) {
 unique(mutations.STAD$icgc_mutation_id[mutations.STAD$icgc_donor_id==donor])
})
names(per_sample_list) <- unique(mutations.STAD$icgc_donor_id)
vcf_list_STAD <- sapply(per_sample_list, function(x) big.vcf[as.character(x)])
names(vcf_list_STAD) <- names(per_sample_list)
vcf_list_STAD <- sapply(vcf_list_STAD, rowRanges)
for (sample in names(vcf_list_STAD)) {
 seqlevels(vcf_list_STAD[[sample]]) <- seqnames(get(ref_genome))[c(as.numeric(seqlevels(vcf_list_STAD[[sample]])[1:22]),25,23,24)]
 vcf_list_STAD[[sample]] <- vcf_list_STAD[[sample]][seqnames(vcf_list_STAD[[sample]])!="chrM"]
 vcf_list_STAD[[sample]] <- vcf_list_STAD[[sample]][seqnames(vcf_list_STAD[[sample]])!="chrY"]
}

all.types <- c(types.full,"INS_A","INS_C","INS_G","INS_T","DEL_A","DEL_C","DEL_G","DEL_T")
sub_list <- sapply(vcf_list_COAD, function(vcf) {
 vcf[width(vcf$REF)==1 & width(unlist(vcf$ALT))==1,]
})
del_list <- sapply(vcf_list_COAD, function(vcf) {
 vcf[width(vcf$REF)==2 & width(unlist(vcf$ALT))==1,]
})
ins_list <- sapply(vcf_list_COAD, function(vcf) {
 vcf[width(vcf$REF)==1 & width(unlist(vcf$ALT))==2,]
})
COAD.mutation.counts = matrix(0,nrow=length(vcf_list_COAD),ncol=length(all.types),dimnames=list(names(vcf_list_COAD),all.types))
for (i in 1:nrow(COAD.mutation.counts)) {
 type_context = type_context(sub_list[[i]], ref_genome)
 counts <- table(type_context)
 for (a in rownames(counts)) {
 tmp = unlist(strsplit(a,split="[>]"))
 inds <- colnames(counts)[counts[a,]>0]
 columns = as.vector(sapply(inds, function(x) paste(substr(x,1,1),"[",a,"]",substr(x,nchar(x),nchar(x)),sep="")))
 COAD.mutation.counts[i,columns] = counts[a,inds]
 }
 COAD.mutation.counts[i,97:100] <- table(substr(unlist(ins_list[[i]]$ALT),2,2))[c("A","C","G","T")]
 COAD.mutation.counts[i,101:104] <- table(substr(del_list[[i]]$REF,2,2))[c("A","C","G","T")]
 print(i)
}
COAD.mutation.counts[is.na(COAD.mutation.counts)] <- 0

sub_list <- sapply(vcf_list_STAD, function(vcf) {
 vcf[width(vcf$REF)==1 & width(unlist(vcf$ALT))==1,]
})
del_list <- sapply(vcf_list_STAD, function(vcf) {
 vcf[width(vcf$REF)==2 & width(unlist(vcf$ALT))==1,]
})
ins_list <- sapply(vcf_list_STAD, function(vcf) {
 vcf[width(vcf$REF)==1 & width(unlist(vcf$ALT))==2,]
})
STAD.mutation.counts = matrix(0,nrow=length(vcf_list_STAD),ncol=length(all.types),dimnames=list(names(vcf_list_STAD),all.types))
for (i in 1:nrow(STAD.mutation.counts)) {
 type_context = type_context(sub_list[[i]], ref_genome)
 counts <- table(type_context)
 for (a in rownames(counts)) {
 tmp = unlist(strsplit(a,split="[>]"))
 inds <- colnames(counts)[counts[a,]>0]
 columns = as.vector(sapply(inds, function(x) paste(substr(x,1,1),"[",a,"]",substr(x,nchar(x),nchar(x)),sep="")))
 STAD.mutation.counts[i,columns] = counts[a,inds]
 }
 STAD.mutation.counts[i,97:100] <- table(substr(unlist(ins_list[[i]]$ALT),2,2))[c("A","C","G","T")]
 STAD.mutation.counts[i,101:104] <- table(substr(del_list[[i]]$REF,2,2))[c("A","C","G","T")]
 print(i)
}
STAD.mutation.counts[is.na(STAD.mutation.counts)] <- 0

Or just upload the prepared data (contains only mutation counts):

load("ICGC data/profiles_and_decomposition.RData")

# Signature extraction

Signature extraction from the whole dataset is performed via Brunet version non-negative matrix factorization using the NMF R package. The number of signatures is chosen as the number of signatures where both residual sum of squares and Akaike Information Criterion values stabilize.

divergence <- function (a,b) {
 return (a * log ( (a+.Machine$double.eps)/(b + .Machine$double.eps)) - a + b)
}
mm <- rbind(COAD.mutation.counts,STAD.mutation.counts)
mut_mat = t(mm) + 0.0001
rss <- NULL; likelihoods <- NULL; divergences <- NULL
for (r in 2:12) {
 res <- NMF::nmf(x=mut_mat,rank=r,seed=123456,method='brunet')
 sigs <- NMF::basis(res)
 cont <- NMF::coef(res)
 rss <- c(rss, sum((t(mm) - sigs%*%cont)**2))
 likelihoods = c(likelihoods,sum(dpois(x=t(mm),lambda=sigs%*%cont,log=T)))
 divergences <- c(divergences,sum(divergence(t(mm),sigs%*%cont)))
}
AIC = 2*c(2:12)*(ncol(mm) + nrow(mm)) - 2*likelihoods

Plot AIC:

df = data.frame(rank=2:12,AIC)
ggplot(df,aes(x=rank,y=AIC)) + geom_point() +
 ggtitle("AIC for selecting the number of signatures") +
 geom_hline(yintercept = AIC[7],linetype = "longdash",colour="red")

Now plot RSS per rank:

df = data.frame(rank=2:12,rss)
ggplot(df,aes(x=rank,y=rss)) + geom_point() +
 ggtitle("RSS for selecting the number of signatures") +
 geom_hline(yintercept = rss[7],linetype = "longdash",colour="red")

This is how the final set of signatures looks like:

res <- NMF::nmf(x=mut_mat,rank=8,seed=123456,method='brunet')
sigs <- NMF::basis(res)
sigs <- sigs[,c(8,1,2,5,4,7,3,6)] # Reorder the signatures
decomposition <- t(NMF::coef(res))
decomposition <- decomposition[,c(8,1,2,5,4,7,3,6)]
colnames(sigs) <- c("Clock-1", "Clock-2", "POLE", "17-like", "MMR-1", "MMR-2", "MMR-3", "SNP")
colnames(decomposition) <- colnames(sigs)
for (i in 1:nrow(decomposition))
 decomposition[i,] = decomposition[i,]/sum(decomposition[i,])
plot_sig_104(sigs,size=12)

Signatures are assigned as follows: Clock-1 (5meC), Clock-2, POLE, 17-like, MMR-1 (20), MMR-2 (15), MMR-3 (21/26), SNP (germline variant contamination).

Obtain microsatellite stability/instability (MSS/MSI) status from UCSC (or rather the Cancer Genome Atlas Clinical Explorer):

icgc.coad <- rownames(COAD.mutation.counts)
icgc.stad <- rownames(STAD.mutation.counts)
coad <- read.delim('ICGC data/COAD/donor.tsv',header=T)
stad <- read.delim('ICGC data/STAD/donor.tsv',header=T)
tcga.coad <- as.character(coad$submitted_donor_id[match(icgc.coad, coad$icgc_donor_id)])
tcga.stad <- as.character(stad$submitted_donor_id[match(icgc.stad, stad$icgc_donor_id)])

mss_coadread <- read.delim('ICGC data/COAD/COADREAD_2015-04-02_ClinicalParameters.txt',header=T)
mss_stad <- read.delim('ICGC data/STAD/STAD_2015-04-02_ClinicalParameters.txt',header=T)
mmr.coad <- icgc.coad[match(intersect(mss_coadread$SampleCode[mss_coadread$MSIstatus=='MSI-H'], tcga.coad),tcga.coad)]
mmr.stad <- icgc.stad[match(intersect(mss_stad$SampleCode[mss_stad$MSIstatus=='MSI-H'], tcga.stad),tcga.stad)]
mmr.ucsc <- c(mmr.stad, mmr.coad)

To assess the distribution of signatures in the data, we generate tSNE plot based on cosine similarity between sample profiles. Circle sizes reflect number of mutations, black outline - microsatellite instability (MSI high), coloured sectors correspond to relative contributions of different signatures. Note that all MSI samples group in one cluster across both datasets.

data <- rbind(COAD.mutation.counts, STAD.mutation.counts)
w <- which(rowSums(data)>0)
data <- data[w,]
cosdist <- function(x,y) {
 x0 <- x/sum(x)
 y0 <- y/sum(y)
 x0 %*% y0 / sqrt(x0%*%x0)/sqrt(y0%*%y0)
}
D <- as.dist(sapply(1:nrow(data), function(i) sapply(1:nrow(data), function(j) 1-cosdist(data[i,],data[j,]) )))
set.seed(1)
t <- tsne(D)
MMR = intersect(rownames(data), mmr.ucsc)
col <- c(RColorBrewer::brewer.pal(8,"Set1")[c(1:2,7:8,3:5)],'darkgrey')
m <- rownames(data) %in% MMR

Visualize the similarity map:

# Plot everything
o1 <- order(rowSums(data[!m,]),decreasing = T)
o2 <- order(rowSums(data[m,]),decreasing = T)
par(bty="n", mar=c(0,0,0,0))
plot(NA,NA, xlab="", ylab="", xlim=c(-20,20), ylim=c(-35,35), xaxt="n", yaxt="n")
corr_scatterpie(t[!m,1][o1], t[!m,2][o1], p=decomposition[w,][!m,][o1,], r=sqrt(rowSums(data)[!m][o1])/75, labels=NA, col=col, lty=0, circles=TRUE, lwd.circle=rep(0.01,sum(!m)),lty.circle=rep(1,sum(!m)), add=TRUE, col.circle = 'white')
corr_scatterpie(t[m,1][o2], t[m,2][o2], p=decomposition[w,][m,][o2,], r=sqrt(rowSums(data)[m][o2])/75, labels=NA, col=col, lty=0, circles=TRUE, lwd.circle=rep(2.5,sum(m)), lty.circle=rep(1,sum(m)), add=TRUE, col.circle = 'black')
par(cex=0.66)
mg14:::.pie(x0=10, y0=27, x=matrix(rep(1,8), nrow=1), r=sqrt(10000)/75, labels=colnames(decomposition), col=col, lty=0, circles=TRUE, add=TRUE)
u <- par("usr")
pr <- (u[2]-u[1])/(u[4]-u[3])
fr <- par("pin")[1]/par("pin")[2]
for(i in c(1,10,100,1000,10000)){
 polygon(20 + cos(seq(0,2*pi, l=100)) * sqrt(i)/75, 25+(1+sin(seq(0,2*pi, l=100))) * sqrt(i)/75 / pr * fr, col=NA)
 if (i>10) text(20, 25 + 2*sqrt(i)/75 / pr * fr + 0.3,labels = as.character(i),cex=0.8)
}
polygon(20 + cos(seq(0,2*pi, l=100)) * sqrt(i)/75, 15+(1+sin(seq(0,2*pi, l=100))) * sqrt(i)/75 / pr * fr, lwd=2.5, col=NA)
text(x = 20,y=24,labels = "Number of mutations")
text(x = 20,y=13,labels = "MSI-H status")

## MMR

Plotting the signature decomposition for MSI samples in both datasets:

p2 <- plot_decomposition(decomposition,mm,intnames=intersect(mmr.ucsc,rownames(STAD.mutation.counts)),col=col) + ggtitle("STAD MSI samples")
p1 <- plot_decomposition(decomposition,mm,intnames=intersect(mmr.ucsc,rownames(COAD.mutation.counts)),col=col) + ggtitle("COAD MSI samples")

Association with MSI status: calculate P-values for relative signature contributions compared with MSI status.

pvals <- NULL
for (i in 1:8)
{
 pvals <- c(pvals, wilcox.test(decomposition[mmr.ucsc,i], decomposition[setdiff(rownames(mm),mmr.ucsc),i], paired=F,alternative='greater')$p.value)
}
names(pvals) = colnames(decomposition)
print(p.adjust(pvals,method = 'bonf')) # Take 0.01 as significance threshold (>10 tests)

MMR-1 seems to be a good indicator of MSI status. Calculate AUC for MMR-1:

library(pROC)
mmr.type <- sapply(rownames(mm), function(x) {
 if (x %in% mmr.ucsc) return("MSI")
 else return("MSS")
})
roc(response = factor(mmr.type), predictor = decomposition[,5])
ci(response = factor(mmr.type), predictor = decomposition[,5])

Boxplots for signature MMR-1, MMR-2, MMR-3 contribution vs MSI status:

df = data.frame(sample = rownames(mm), mmr.type, decomposition[,c(5:7)])
colnames(df)[3:5] = colnames(decomposition)[c(5:7)]
df = melt(df)

p1 <- ggplot(df, aes(x=mmr.type,y=value,fill=variable)) + geom_boxplot(width=0.4,size=0.1,outlier.size=0.5) +
 facet_grid(. ~ variable) + scale_fill_manual(values=col[c(5:7)]) +
 ylab("Relative contribution") + xlab("") + guides(fill=F) + theme_bw() +
 theme(text = element_text(family='ArialMT'),
 strip.text.x = element_text(size=16),
 axis.title.y = element_text(size=16),
 axis.text.x = element_text(size=10),
 axis.text.y = element_text(size=10),
 panel.grid = element_blank(),
 panel.border = element_rect(size = 0.1),
 strip.background = element_blank())
p1

As we can see from the signature plot, all indels are associated with MMR-1; to confirm this, the absolute contribution of MMR signatures vs the number of indels was plotted:

new.cont.mat <- t(decomposition)
for (i in 1:ncol(new.cont.mat))
 new.cont.mat[,i] <- new.cont.mat[,i] * rowSums(mm)[i]
df = data.frame(sample=rownames(mm),ind=rowSums(mm[,97:104]),mmr1=new.cont.mat[5,],mmr2=new.cont.mat[6,],mmr3=new.cont.mat[7,])
df <- melt(df, id=c('sample','ind'))
p <- ggplot() + geom_point(data = df[df$variable=='mmr3',], aes(x=ind,y=value), col=col[7],size=0.5) +
 geom_point(data = df[df$variable=='mmr2',], aes(x=ind,y=value), col=col[6],size=0.5) +
 geom_point(data = df[df$variable=='mmr1',], aes(x=ind,y=value), col=col[5],size=0.5) +
 theme_bw() +
 theme(text = element_text(family='ArialMT'),
 strip.text.x = element_text(size=16),
 axis.title.y = element_text(size=16),
 axis.text.x = element_text(size=10),
 axis.text.y = element_text(size=10),
 panel.grid = element_blank(),
 panel.border = element_rect(size = 0.1),
 strip.background = element_blank())
 #xlab('Number of 1 bp indels') + ylab('Number of mutations assigned to a signature') +
p

To see the unusual contribution of the Clock-1 signature, we plot the average fold change (log scale) in the number of mutations assigned to different signatures in MSI samples and MSS samples:

df = data.frame(sigs = colnames(decomposition), val=log10(rowMeans(new.cont.mat[,mmr.ucsc]) / rowMeans(new.cont.mat[,setdiff(rownames(mm),mmr.ucsc)])))
p <- ggplot(data=df, aes(x=factor(sigs,levels=rev(colnames(decomposition))),y=val)) +
 geom_bar(stat='identity', fill=rev(col)[c(6,1:5,7:8)]) +
 coord_flip() +
 theme_bw() +
 theme(text = element_text(family='ArialMT'),
 strip.text.x = element_text(size=16),
 axis.title.y = element_text(size=16),
 axis.text.x = element_text(size=10),
 axis.text.y = element_text(size=10),
 panel.grid = element_blank(),
 panel.border = element_rect(size = 0.1),
 strip.background = element_blank()) +
 ylab('no. mutations in MSI / no. mutations in MSS') + xlab('Signatures') +
 scale_y_continuous(breaks=c(0,1,2),labels=c(1,10,100))
p

Relative contributions for MMR deficient samples:

p1 <- plot_decomposition(decomposition,decomposition,intersect(rownames(COAD.mutation.counts),mmr.ucsc),col,axis.size=20)
p2 <- plot_decomposition(decomposition,decomposition,intersect(rownames(STAD.mutation.counts),mmr.ucsc),col,size=4, axis.size=20)

Absolute contributions for all the samples across both cancer datasets:

p1 <- plot_decomposition(decomposition,mm,rownames(COAD.mutation.counts),col,size=2, axis.size=14)
p2 <- plot_decomposition(decomposition,mm,rownames(STAD.mutation.counts),col, size=2, axis.size=14)

## Amount of indels

Plotting the amount of indels per sample across both cancer datasets:

indels <- rowSums(mm[,97:104])
cohort <- ifelse(rownames(mm)[indels>10] %in% rownames(COAD.mutation.counts),"COAD","STAD")
df <- data.frame(indelcount=indels[indels>10],cohort,sample=rownames(mm)[indels>10])
ggplot(data=df, aes(x=factor(sample,levels=names(sort(indels))),y=indelcount,fill=cohort)) + geom_bar(stat="identity") + facet_grid(. ~ cohort,scales = "free")

Identify all the homopolymers of length 4 to 55 (max length observed) in human exome:

1. Create homopolymer library:

homopolymer_pool_length_4to55 <- lapply(seq(4,55),function(y) lapply(c("A","C","G","T"),function(x)
 if (x=="A") {paste0("B",paste(rep(x,y),collapse=""),"B")
 }
 else
 if (x=="T") {paste0("V",paste(rep(x,y),collapse=""),"V")
 }
 else
 if (x=="G") {paste0("H",paste(rep(x,y),collapse=""),"H")
 }
 else
 if (x=="C") {paste0("D",paste(rep(x,y),collapse=""),"D")
 }
))
homopolymers <- unlist(homopolymer_pool_length_4to55)
pattern_all <- DNAStringSet(homopolymers)

1. Find all homopolymers in the genome:

hits_all <- list()
for (i in 1:23) {
 maskMotif(a[[i]], "N") -> masked
 hits_all[[i]] <- sapply(pattern_all,
 matchPattern,
 subject=masked,
 fixed=F)
}
lengths <- lapply(hits_all, function(l) sapply(l,length))
hits_all <- lapply(1:length(hits_all), function(i) hits_all[[i]][which(lengths[[i]]>0)])
nonzero_lengths <- lapply(hits_all, function(chr) sapply(chr, length)) # numbers of homopolymers on each chromosome for non empty classes only
names(hits_all) <- seqlevels(gr)

1. Create a dataframe with their coordinates and contexts:

sites.gr <- lapply(1:23, function(i) do.call("c",lapply(lapply(hits_all[[i]], as, "IRanges"),
 GRanges,seqnames=names(hits_all)[i])))
for (j in 1:23) {
 sites.gr[[j]]$pattern.searched <- rep(as.character(pattern_all), lengths[[j]])
 sites.gr[[j]]$motif.found <- unlist(lapply(hits_all[[j]], as.character))
 sites.gr[[j]]$pattern.length <- unlist(lapply(hits_all[[j]], width)) # includes the two flanking bases
 sites.gr[[j]]$homopolymer.length <- (sites.gr[[j]]$pattern.length)-2 # remove 2 bases, 5' and 3' are not part of homopolymer
}
sites.gr_all <- do.call("c",sites.gr)
genome(sites.gr_all) <- "hg19" # add genome info

1. Intersect with well-covered part of the human exome:

exomranges <- GRanges(seqnames=exome$V1,ranges=IRanges(start = exome$V2,end=exome$V3))
hits <- findOverlaps(exomranges,sites.gr_all,minoverlap = 2)
sites.gr_all <- sites.gr_all[sort(unique(subjectHits(hits)))]
all_sites <- as.data.frame(sites.gr_all) # make a data frame

1. Check the amount of 1bp indels in homopolymers in MSI samples in both cancer cohorts (requires lists of vcf objects for both cohorts):

# Upload the vcf files for both cohorts
indel_list_STAD <- sapply(vcf_list_STAD, function(vcf) vcf[abs(width(vcf$REF)-width(unlist(vcf$ALT)))==1 & (width(vcf$REF)==1 | width(unlist(vcf$ALT))==1),])
indel_list_COAD <- sapply(vcf_list_COAD, function(vcf) vcf[abs(width(vcf$REF)-width(unlist(vcf$ALT)))==1 & (width(vcf$REF)==1 | width(unlist(vcf$ALT))==1),])
for (k in 1:length(indel_list_STAD)) {
 genome(indel_list_STAD[[k]]) <- "hg19"
 indel_list_STAD[[k]]$insertion <- width(unlist(indel_list_STAD[[k]]$ALT))-1
 indel_list_STAD[[k]]$deletion <- width(indel_list_STAD[[k]]$REF)-1
}
for (k in 1:length(indel_list_COAD)) {
 genome(indel_list_COAD[[k]]) <- "hg19"
 indel_list_COAD[[k]]$insertion <- width(unlist(indel_list_COAD[[k]]$ALT))-1
 indel_list_COAD[[k]]$deletion <- width(indel_list_COAD[[k]]$REF)-1
}
indels.in.hp.stad <- vector("numeric",length(indel_list_STAD))
for (i in 1:length(indel_list_STAD)) {
 x <- subsetByOverlaps(indel_list_STAD[[i]],sites.gr_all)
 indels.in.hp.stad[i] <- nrow(as.data.frame(x))
}
names(indels.in.hp.stad) <- names(indel_list_STAD)
indels.in.hp.coad <- vector("numeric",length(indel_list_COAD))
for (i in 1:length(indel_list_COAD)) {
 x <- subsetByOverlaps(indel_list_COAD[[i]],sites.gr_all)
 indels.in.hp.coad[i] <- nrow(as.data.frame(x))
}
names(indels.in.hp.coad) <- names(indel_list_COAD)

1. Plot indels in homopolymers:

df <- data.frame(sample=rownames(STAD.mutation.counts), Non.HP.indels = rowSums(STAD.mutation.counts[,97:104])-indels.in.hp.stad[rownames(STAD.mutation.counts)], HP.indels = indels.in.hp.stad[rownames(STAD.mutation.counts)])
df = melt(df,id.vars="sample")
p1 <- ggplot(data=df[df$sample %in% mmr.ucsc,], aes(x=factor(sample, levels = df$sample[order(rowSums(STAD.mutation.counts[,97:104]), decreasing = F)]),y=value,fill=variable)) + geom_bar(stat="identity") +
 theme_bw() +
 theme(axis.text.x = element_text(angle = 90,vjust=0.5, hjust=0, size=8, family='ArialMT'),
 axis.text.y = element_text(size=8, family='ArialMT'),
 legend.title=element_blank(),
 axis.title = element_text(size=8, family='ArialMT'),
 legend.text = element_text(size=8,family='ArialMT'),
 title = element_text(size=10, family='ArialMT', face='bold'),
 panel.border = element_rect(colour='white'),
 panel.grid = element_blank()) +
 scale_fill_discrete(labels=c("Indels not in HP","Indels in HP")) + xlab("MSI sample") +
 ylab("1bp indel counts") + ggtitle("1 bp indels in homopolymers in STAD samples with MSI")
p1

df <- data.frame(sample=rownames(COAD.mutation.counts), Non.HP.indels = rowSums(COAD.mutation.counts[,97:104])-indels.in.hp.coad[rownames(COAD.mutation.counts)], HP.indels = indels.in.hp.coad[rownames(COAD.mutation.counts)])
df = melt(df,id.vars="sample")
p2 <- ggplot(data=df[df$sample %in% mmr.ucsc,], aes(x=factor(sample, levels = df$sample[order(rowSums(COAD.mutation.counts[,97:104]), decreasing = F)]),y=value,fill=variable)) + geom_bar(stat="identity") +
 theme_bw() +
 theme(axis.text.x = element_text(angle = 90,vjust=0.5, hjust=0, size=8, family='ArialMT'),
 axis.text.y = element_text(size=8, family='ArialMT'),
 legend.title=element_blank(),
 axis.title = element_text(size=8, family='ArialMT'),
 legend.text = element_text(size=8,family='ArialMT'),
 title = element_text(size=10, family='ArialMT', face='bold'),
 panel.border = element_rect(colour='white'),
 panel.grid = element_blank()) +
 scale_fill_discrete(labels=c("Indels not in HP","Indels in HP")) + xlab("MSI sample") +
 ylab("1bp indel counts") + ggtitle("1 bp indels in homopolymers in COAD samples with MSI")
p2

## More about signature contributions

Create boxplots of relative contributions in MSI and MSS samples for all signatures simultaneously:

mmr.type <- ifelse(rownames(mm) %in% mmr.ucsc, "MSI", "MSS")
df = data.frame(decomposition,mmr.type, sample = rownames(mm))
df2 <- melt(df,id.vars = c("mmr.type","sample"))
ggplot(data=df2, aes(x=factor(mmr.type),y=value,fill=variable)) + geom_boxplot() + facet_grid(. ~ variable,scales = "free")

Absolute contributions:

mmr.type <- ifelse(rownames(mm) %in% mmr.ucsc, "MSI", "MSS")
df = data.frame(log(t(round(new.cont.mat))),mmr.type, sample = rownames(mm))
df2 <- melt(df,id.vars = c("mmr.type","sample"))
ggplot(data=df2, aes(x=factor(mmr.type),y=value,fill=variable)) + geom_boxplot() + facet_grid(. ~ variable,scales = "free")

## Similarities

We calculate cosine similarities between COSMIC signatures and *de novo* signatures identified in the COAD and STAD cancer sample set; and also between *C. elegans* signatures and all of the abovementioned. The cosine similarity score measures the cosine of an angle between two vectors:

$$Sim(a,b)=\frac{<a,b>}{||a||*||b||}$$

cosine <- function(x,y) {
 x %*% y / sqrt(sum(x**2)) / sqrt(sum(y**2))
}

The higher the similarity, the closer the vectors are to each other.

Similarities between *de novo* signatures and *C. elegans* derived *mlh-1*, *pms-2* and *pole-4;pms-2* mutational patterns:

tmp <- matrix(0,nrow=8,ncol=3,dimnames=list(colnames(sigs),rownames(learned.sigs)[c(2,3,5)]))
for (i in c(1:8))
 for (j in c(1:3)) {
 tmp[i,j] <- round(cosine(sigs[1:96,i],learned.sigs.exome[j,1:96]),2)
 }
print(tmp)

Similarities between *de novo* signatures and COSMIC cancer signatures:

tmp <- matrix(0,nrow=8,ncol=30,dimnames=list(colnames(sigs),colnames(cancer_signatures)))
for (i in c(1:8))
 for (j in 1:30) {
 tmp[i,j] <- round(cosine(sigs[1:96,i],cancer_signatures_adj[1:96,j]),2)
 }
print(tmp)

## Age correlation

Testing if contribution of any signature correlates with age. Correlation with both relative and absolute contributions is very low.

Correlation with relative contributions:

donorinfo <- rbind(read.delim("ICGC data/COAD/donor.tsv"),read.delim("ICGC data/STAD/donor.tsv"))
tmp <- vector("numeric",8)
for (i in 1:8)
 tmp[i] <- (cor(donorinfo$donor_age_at_diagnosis[match(rownames(mm)[-c( 232, 484, 494)],donorinfo$icgc_donor_id)],decomposition[-c(232,484,494),i],method='spearman'))
names(tmp) <- colnames(sigs)
print(tmp)

Correlation with absolute contributions:

tmp <- vector("numeric",8)
for (i in 1:8)
 tmp[i] <- (cor(donorinfo$donor_age_at_diagnosis[match(rownames(mm)[-c( 232, 484, 494)],donorinfo$icgc_donor_id)],new.cont.mat[i,-c(232,484,494)],method='spearman'))
names(tmp) <- colnames(sigs)
print(tmp)

## Average profiles

Plot averaged profiles of MSI samples in COAD and STAD datasets. Their cosine similarity shows that they are nearly identical.

p1 <- plot_profiles_104(t(mm[intersect(rownames(STAD.mutation.counts),mmr.ucsc),]), boxplot = T, normalize = T, size=14)
p2 <- plot_profiles_104(t(mm[intersect(rownames(COAD.mutation.counts),mmr.ucsc),]), boxplot = T, normalize = T, size=14)
p1
p2

print(cosine(colMeans(mm[intersect(rownames(STAD.mutation.counts),mmr.ucsc),]),colMeans(mm[intersect(rownames(COAD.mutation.counts),mmr.ucsc),])))

# Cosine similarity simulations for signature comparison

Some necessary functions: **cosine** – returns cosine similarity score between two vectors, **cosineM** calculates a matrix of similarities between columns of a matrix, **poisI** calculates Fisher information matrix for Poisson model using design matrix *X*, coefficient vector *theta* and outcome vector *Y*.

cosine <- function(x,y) {
 return(sum(x * y) / sqrt(sum(x**2)) / sqrt(sum(y**2)))
}
cosineM <- function(X) {
 return(sapply(1:ncol(X), function(i) sapply(1:ncol(X), function(j) cosine(X[,i], X[,j]))))
}
poisI <- function(X, theta, Y){
 lambda <- as.numeric(X %*% theta)
 t(Y/lambda^2 * X) %*% X
} # Fisher information matrix for Poisson model

Generate uniformly distributed profiles in positive cone and calculate the distribution of similarity scores. Its mean is close to 0.75, and it’s 95-% quantile is around 0.80.

maxs <- sample(1:5000, 1000, replace=T)
X <- sapply(1:1000, function(x) runif(n = 104, min = 0, max=maxs[x]))
X <- apply(X,2,function(y) y/sum(y))
XM <- cosineM(X)
df = data.frame(x = 1:length(XM[upper.tri(XM)]), val = XM[upper.tri(XM)])
p <- ggplot(data = df, aes(val)) +
 geom_histogram(binwidth = 0.005, col='black', fill='white') +
 theme_bw() + theme(panel.grid = element_blank(), text = element_text(family='ArialMT'), panel.border = element_rect(colour='white')) +
 geom_vline(xintercept = quantile(as.vector(XM[upper.tri(XM)]), 0.95), col='red', linetype='dashed') +
 ggtitle('Distribution of similarities between uniform random vectors from positive cone') +
 xlab('Cosine similarity score') + ylab('Frequency') + xlim(c(0,1))
ggsave(plot=p, height=4,width=7, filename = '~/Documents/uniform_sim.pdf', useDingbats=FALSE)

## Distribution of angles in COSMIC signatures

Plotting the histogram and heatmap for similarity scores between COSMIC signatures:

cosmic <- cosineM(cancer_signatures)
hist(cosmic[upper.tri(cosmic)], prob=T, breaks=20, main='Similarities between COSMIC signatures', xlab = 'Cosine similarity')
lines(density(cosmic[upper.tri(cosmic)], adjust=2), lty="dotted")

# Heatmap
image.plot(cosmic)
for (x in 1:ncol(cosmic))
 for (y in 1:ncol(cosmic))
 text((x-1)/(ncol(cosmic)-1), (y-1)/(ncol(cosmic)-1), sprintf("%0.2f", cosmic[x,y]))
# ggplot histogram
df = data.frame(x = 1:length(cosmic[upper.tri(cosmic)]), val=cosmic[upper.tri(cosmic)])
p <- ggplot(data = df, aes(val)) +
 geom_histogram(binwidth = 0.05, col='black', fill='white') +
 theme_bw() + theme(panel.grid = element_blank(), text = element_text(family='ArialMT'), panel.border = element_rect(colour='white')) +
 geom_vline(xintercept = 0.8, col='red', linetype='dashed') +
 ggtitle('Distribution of similarities between COSMIC cancer signatures') +
 xlab('Cosine similarity score') + ylab('Frequency') + xlim(c(0,1))
ggsave(plot=p, height=4,width=7, filename = '~/Documents/cosmic_sim.pdf', useDingbats=FALSE)

## Similarity between *C. elegans* mutational patterns and human *de novo* signatures

Now we draw a signature from bootstrapped COADSTAD signatures, and compare it to a random draw from a confidence interval for *C. elegans* signature.

Calculate again the *de novo* signatures:

load('ICGC data/profiles_and_decomposition.RData')
mm <- rbind(COAD.mutation.counts,STAD.mutation.counts)
mut_mat = t(mm) + 0.0001
res <- nmf(mut_mat,rank=8,seed=123456,method='brunet')
defsigs <- NMF::basis(res)
defsigs <- defsigs[,c(8,1,2,5,4,7,3,6)]
colnames(defsigs) <- c("Clock-1", "Clock-2", "POLE", "17-like", "MMR-1", "MMR-2", "MMR-3", "SNP")

Bootstrapping across COAD/STAD samples:

sigs = list()
for (i in 1:505) {
 mm = rbind(COAD.mutation.counts,STAD.mutation.counts)
 mut_mat <- t(mm[-i,]) + 0.0001
 res <- nmf(mut_mat, rank = 8, method='brunet')
 sigs[[i]] <- NMF::basis(res)
}

Now we calculate confidence intervals for the *C. elegans* mutational patterns:

load('C.elegans data/Learned_signatures_indel.RData')
learned.sigs <- nmSolve(worm.donor.mut.mat[,1:96],small.X,maxIter=10000, tol = 1e-06, div.err = 1e-10)
Y = worm.donor.mut.mat
mut_matrix_lower <- matrix(NA,nrow=96,ncol=nrow(learned.sigs),dimnames=list(colnames(learned.sigs)[1:96],row.names(learned.sigs)))
mut_matrix_upper <- matrix(NA,nrow=96,ncol=nrow(learned.sigs),dimnames=list(colnames(learned.sigs)[1:96],row.names(learned.sigs)))
library(MASS)
for (i in 1:96) {
 cov.mat <- poisI(as.matrix(small.X),learned.sigs[,i],Y[,i])
 to.keep <- colnames(cov.mat)[which(diag(cov.mat)!=0)]
 if (length(to.keep)!=0) {
 cov.mat <- cov.mat[to.keep,to.keep]
 SVD <- svd(cov.mat)$d
 if (length(which(SVD<1e-6))>0)
 cov.mat <- ginv(cov.mat)
 if (length(which(SVD<1e-6))==0)
 cov.mat <- solve(cov.mat)
 row.names(cov.mat) <- to.keep
 colnames(cov.mat) <- to.keep
 sterr <- sqrt(diag(cov.mat))
 mut_matrix_lower[i,to.keep] <- learned.sigs[to.keep,i] - sterr * qt(0.975,30)
 mut_matrix_upper[i,to.keep] <- learned.sigs[to.keep,i] + sterr * qt(0.975,30)
 mut_matrix_lower[i,to.keep][mut_matrix_lower[i,to.keep]<0] <- 0
 }
}

Adjusting for trinucleotide difference:

load('exome.RData')
learned.sigs.exome <- learned.sigs[,1:96]
mut_matrix_lower.exome <- t(mut_matrix_lower)
mut_matrix_upper.exome <- t(mut_matrix_upper)
for (i in 1:nrow(learned.sigs.exome)) {
 learned.sigs.exome[i,] <- learned.sigs.exome[i,] / trinucleotide.freq.factor.ex[types]
 mut_matrix_lower.exome[i,1:96] <- mut_matrix_lower.exome[i,1:96] / trinucleotide.freq.factor.ex[types] # + learned.sigs.exome[i,1:96] - learned.sigs[i,1:96]
 mut_matrix_upper.exome[i,1:96] <- mut_matrix_upper.exome[i,1:96] / trinucleotide.freq.factor.ex[types] # + learned.sigs.exome[i,1:96] - learned.sigs[i,1:96]
}
mut_matrix_lower.exome[mut_matrix_lower.exome<0] <- 0
# plot signatures with CIs
plot_96_profile_CI(mut_matrix = t(learned.sigs[c(2:3,5),1:96]), mut_matrix_lower = mut_matrix_lower[,c(2:3,5)], mut_matrix_upper = mut_matrix_upper[,c(2:3,5)], CI=TRUE)
plot_96_profile_CI(mut_matrix = t(learned.sigs.exome[c(2:3,5),1:96]), mut_matrix_lower = t(mut_matrix_lower.exome[c(2:3,5),]), mut_matrix_upper = t(mut_matrix_upper.exome[c(2:3,5),]), CI=TRUE)

Now we can draw *C. elegans* patterns randomly from their confidence intervals and compare to bootstrapped *de novo* signatures.

Similarities for fixed signatures:

cosine(learned.sigs.exome[3,1:96],defsigs[1:96,5]) # # 0.85
cosine(learned.sigs.exome[2,1:96],defsigs[1:96,5]) # # 0.81
cosine(learned.sigs.exome[3,-c(35,39,43,47,97:104)],defsigs[-c(35,39,43,47,97:104),5]) # 0.92
cosine(learned.sigs.exome[2,-c(35,39,43,47,97:104)],defsigs[-c(35,39,43,47,97:104),5]) # 0.90

Drawing random signatures:

random_signature <- function(sig, sig_low, sig_up) {
 res <- sapply(1:length(sig), function(i) rnorm(1,mean=sig[i],sd=(sig_up[i]-sig[i])/2))
 res[res<0] <- 0
 res[is.nan(res)] <- sig[is.nan(res)]
 return(res)
}

*pole4;pms-2* double mutant versus single mutant signatures:

a1 <- sapply(1:1000, function(s) cosine(random_signature(sig=learned.sigs[2,1:96],sig_low = mut_matrix_lower[,2],sig_up = mut_matrix_upper[,2]),
 random_signature(sig=learned.sigs[5,1:96],sig_low = mut_matrix_lower[,5],sig_up = mut_matrix_upper[,5])))
a2 <- sapply(1:1000, function(s) cosine(random_signature(sig=learned.sigs[3,1:96],sig_low = mut_matrix_lower[,3],sig_up = mut_matrix_upper[,3]),
 random_signature(sig=learned.sigs[5,1:96],sig_low = mut_matrix_lower[,5],sig_up = mut_matrix_upper[,5])))
a3 <- sapply(1:1000, function(s) cosine(random_signature(sig=learned.sigs[2,1:96],sig_low = mut_matrix_lower[1:96,2],sig_up = mut_matrix_upper[1:96,2]),
 random_signature(sig=learned.sigs[3,1:96],sig_low = mut_matrix_lower[1:96,3],sig_up = mut_matrix_upper[1:96,3])))

df = data.frame(x = 1:length(a1), a1, a2, a3)
df = melt(df, id.vars = "x")
levels(df$variable) <- c('mlh-1 vs pole-4;pms-2', 'pms-2 vs pole-4;pms-2', 'mlh-1 vs pms-2')
df2 <- data.frame(cos = c(cosine(learned.sigs[2,1:96],learned.sigs[5,1:96]),
 cosine(learned.sigs[3,1:96],learned.sigs[5,1:96]),
 cosine(learned.sigs[2,1:96],learned.sigs[3,1:96])),
 variable = c('mlh-1 vs pole-4;pms-2', 'pms-2 vs pole-4;pms-2', 'mlh-1 vs pms-2'))
p <- ggplot(data = df, aes(value)) +
 geom_histogram(binwidth = 0.01, col='black', fill='white') +
 facet_grid(variable ~ ., scales='fixed') +
 theme_bw() + theme(panel.grid = element_blank(), text = element_text(family='ArialMT'), strip.background = element_rect(colour='white', fill='white'),
 panel.border = element_rect(colour = 'white'), strip.text = element_text(size=12)) +
 geom_vline(data=df2, aes(xintercept=cos), col='red', linetype='dashed') +
 ggtitle('Distribution of similarities between C. elegans signatures') +
 xlab('Cosine similarity score') + ylab('Frequency') + xlim(c(0,1))
p

Humanized *mlh-1* and *pms-2* vs MMR-1:

a1 <- sapply(1:505, function(s) cosine(random_signature(sig=learned.sigs.exome[3,1:96],sig_low = mut_matrix_lower.exome[3,],sig_up = mut_matrix_upper.exome[3,]),jkset[[5]][-c(97:104),s]))
a2 <- sapply(1:505, function(s) cosine(random_signature(sig=learned.sigs.exome[3,c(1:96)[-c(35,39,43,47)]],sig_low = mut_matrix_lower.exome[3,-c(35,39,43,47)],
 sig_up = mut_matrix_upper.exome[3,-c(35,39,43,47)]),jkset[[5]][-c(35,39,43,47,97:104),s]))
b1 <- sapply(1:505, function(s) cosine(random_signature(sig=learned.sigs.exome[2,1:96],sig_low = mut_matrix_lower.exome[3,],
 sig_up = mut_matrix_upper.exome[3,]),jkset[[5]][-c(97:104),s]))
b2 <- sapply(1:505, function(s) cosine(random_signature(sig=learned.sigs.exome[2,c(1:96)[-c(35,39,43,47)]],sig_low = mut_matrix_lower.exome[3,-c(35,39,43,47)],
 sig_up = mut_matrix_upper.exome[3,-c(35,39,43,47)]),jkset[[5]][-c(35,39,43,47,97:104),s]))


a1 <- sapply(1:505, function(s) cosine(learned.sigs.exome[3,1:96],jkset[[5]][-c(97:104),s]))
a2 <- sapply(1:505, function(s) cosine(learned.sigs.exome[3,c(1:96)[-c(35,39,43,47)]],jkset[[5]][-c(35,39,43,47,97:104),s]))
b1 <- sapply(1:505, function(s) cosine(learned.sigs.exome[2,1:96],jkset[[5]][-c(97:104),s]))
b2 <- sapply(1:505, function(s) cosine(learned.sigs.exome[2,c(1:96)[-c(35,39,43,47)]],jkset[[5]][-c(35,39,43,47,97:104),s]))

Plot for humanized *pms-2* versus MMR-1 with and without C>Ts at CpG sites:

df = data.frame(x = 1:length(a1), a1, a2)
df = melt(df, id.vars = "x")
levels(df$variable) <- c('Full signatures', 'Without C>T at CpG sites')
df2 <- data.frame(cos = c(cosine(learned.sigs.exome[3,1:96],defsigs[1:96,5]),
 cosine(learned.sigs.exome[3,-c(35,39,43,47,97:104)],defsigs[-c(35,39,43,47,97:104),5])),
 variable =c('Full signatures', 'Without C>T at CpG sites'))
p <- ggplot(data = df, aes(value)) +
 geom_histogram(binwidth = 0.01, col='black', fill='white') +
 facet_grid(. ~ variable, scales='fixed') +
 theme_bw() + theme(panel.grid = element_blank(), text = element_text(family='ArialMT'), strip.background = element_rect(colour='white', fill='white'),
 panel.border = element_rect(colour = 'white'), strip.text = element_text(size=12)) +
 geom_vline(data=df2, aes(xintercept = cos), col='red', linetype='dashed') +
 ggtitle('Distribution of similarities between pms-2 and MMR-1 signatures') +
 xlab('Cosine similarity score') + ylab('Frequency') + xlim(c(0,1))
p

Plot for humanized *mlh-1* versus MMR-1 with and without C>Ts at CpG sites:

df = data.frame(x = 1:length(a1), b1, b2)
df = melt(df, id.vars = "x")
levels(df$variable) <- c('Full signatures', 'Without C>T at CpG sites')
df2 <- data.frame(cos = c(cosine(learned.sigs.exome[2,1:96],defsigs[1:96,5]),
 cosine(learned.sigs.exome[2,-c(35,39,43,47,97:104)],defsigs[-c(35,39,43,47,97:104),5])),
 variable =c('Full signatures', 'Without C>T at CpG sites'))
p <- ggplot(data = df, aes(value)) +
 geom_histogram(binwidth = 0.01, col='black', fill='white') +
 facet_grid(. ~ variable, scales='fixed') +
 theme_bw() + theme(panel.grid = element_blank(), text = element_text(family='ArialMT'), strip.background = element_rect(colour='white', fill='white'),
 panel.border = element_rect(colour = 'white'), strip.text = element_text(size=12)) +
 geom_vline(data=df2, aes(xintercept = cos), col='red', linetype='dashed') +
 ggtitle('Distribution of similarities between mlh-1 and MMR-1 signatures') +
 xlab('Cosine similarity score') + ylab('Frequency') + xlim(c(0,1))
p

# SNP contamination signature

Checking the presence of human population SNPs identified in the 1000 genome project in COAD/STAD samples. This analysis provides evidence for *de novo* signature 8 representing SNP contamination.

Obtain the SNPs:

library('SNPlocs.Hsapiens.dbSNP144.GRCh37')
snps <- SNPlocs.Hsapiens.dbSNP144.GRCh37

Finding the substitutions intersecting with SNPs in COAD variants:

regs <- sapply(vcf_list_COAD, function(vcf) {
 vcf[width(vcf$REF)==1 & width(unlist(vcf$ALT))==1,]
})
# Adjust chromosome names
for (sample in names(regs)) {
 seqlevels(regs[[sample]]) <- substr(seqlevels(regs[[sample]]),4,nchar(seqlevels(regs[[sample]])))
}
# Intersect SNPs and substitutions
snps_in_regs <- list()
for (i in seq_along(vcf_list_COAD)) {
 genome(regs[[i]]) <- "GRCh37.p13"
 snps_in_regs[[i]] <- snpsByOverlaps(snps,regs[[i]],minoverlap=1)
 inds <- match(pos(snps_in_regs[[i]]),start(regs[[i]]))
 alts <- as.character(unlist(regs[[i]][inds]$ALT))
 snpalts <- as.character(IUPAC_CODE_MAP[snps_in_regs[[i]]$alleles_as_ambig])
 if (length(alts)==0) next
 snps_in_regs[[i]] <- snps_in_regs[[i]][sapply(1:length(inds), function(j) {
 grepl(alts[j],snpalts[j])
 })]
}
names(snps_in_regs) <- names(regs)

Finding the substitutions intersecting with SNPs in STAD variants:

STregs <- sapply(vcf_list_STAD, function(vcf) {
 vcf[width(vcf$REF)==1 & width(unlist(vcf$ALT))==1,]
})
# Intersect SNPs and substitutions
STsnps_in_regs <- list()
for (i in seq_along(vcf_list_STAD)) {
 seqlevels(STregs[[i]]) <- substr(seqlevels(STregs[[i]]),4,nchar(seqlevels(STregs[[i]])))
 genome(STregs[[i]]) <- "GRCh37.p13"
 STsnps_in_regs[[i]] <- snpsByOverlaps(snps,STregs[[i]],minoverlap=1)
 print(i)
 inds <- match(pos(STsnps_in_regs[[i]]),start(STregs[[i]]))
 alts <- as.character(unlist(STregs[[i]][inds]$ALT))
 snpalts <- as.character(IUPAC_CODE_MAP[STsnps_in_regs[[i]]$alleles_as_ambig])
 if (length(alts)==0) next
 STsnps_in_regs[[i]] <- STsnps_in_regs[[i]][sapply(1:length(inds), function(j) {
 grepl(alts[j],snpalts[j])
 })]
}
names(STsnps_in_regs) <- names(STregs)

Plotting the fraction of potential SNPs to all variants per sample:

hist( sapply(STsnps_in_regs,length) / sapply(STregs,length), breaks=20)

Obtain coding and non-coding SNPs from 1000 Genomes project (<http://www.internationalgenome.org/category/vcf/>):

coding_vcf <- readVcf("ALL.wgs.integrated_phase1_release_v3_coding_annotation.20101123.snps_indels.sites.vcf")
non_coding_vcf <- readVcf("ALL.wgs.integrated_phase1_release_v3_noncoding_annotation_20120330.20101123.snps_indels_sv.sites.vcf.gz")

Counting the number of coding SNPs:

regs <- c(regs, STregs)
cod_snps_by_overlaps <- sapply(regs, function(reg) {
 tmp <- findOverlaps(granges(coding_vcf), reg)
 alts <- as.character(unlist(reg[subjectHits(tmp)]$ALT))
 snpalts <- as.character(unlist(granges(coding_vcf)$ALT))[queryHits(tmp)]
 tmp[alts==snpalts]
})
#non_cod_snps_by_overlaps <- sapply(regs, function(reg) findOverlaps(non_coding_vcf, reg))
counts <- sapply(cod_snps_by_overlaps, function(x)
 return(c(length(grep(":synonymous:",unlist(info(coding_vcf[queryHits(x)])$VA))),
 length(grep(":nonsynonymous:",unlist(info(coding_vcf[queryHits(x)])$VA))))))

Calculating the ratio of nonsynonymous to synonymous changes:

length(grep(":nonsynonymous:",unlist(info(coding_vcf)$VA))) / length(grep(":synonymous:",unlist(info(coding_vcf)$VA)))

Next, we check the samples with presence of signature 8. The ratio of synonymous to non-synonymous changes in them seems to be too high for a cancer sample and rather reflect germline variants.

suspects <- rownames(decomposition)[decomposition[,8]>0.3]
barplot(counts[2,suspects[counts[1,suspects]!=0]] / (counts[1,suspects[counts[1,suspects]!=0]]))

# Homopolymer analysis in *C. elegans*

First, we define homopolymer sequences:

motif_6bp_HP <- DNAStringSet(c("BAAAAAAB", "VTTTTTTV", "HGGGGGGH", "DCCCCCCD"))
homopolymer_pool_length_4to35 <- lapply(seq(4,35),function(y) lapply(c("A","C","G","T"),function(x)
 if (x=="A") {paste0("B",paste(rep(x,y),collapse=""),"B")
 }
 else
 if (x=="T") {paste0("V",paste(rep(x,y),collapse=""),"V")
 }
 else
 if (x=="G") {paste0("H",paste(rep(x,y),collapse=""),"H")
 }
 else
 if (x=="C") {paste0("D",paste(rep(x,y),collapse=""),"D")
 }
))
whomopolymers <- unlist(homopolymer_pool_length_4to35)
list(whomopolymers)
# turn into DNA stringset #
pattern_all <- DNAStringSet(whomopolymers)

Finding homopolymers in each chromosome:

whits_all <- lapply(WBcel235, function(chr) sapply(pattern_all, matchPattern, subject=chr, fixed=FALSE))
wlengths <- lapply(whits_all, function(chr) sapply(chr, length)) # numbers of homopolymers on each chromosome
wnonzero_hits_all <- lapply(1:7, function(i) whits_all[[i]][which(wlengths[[i]]>0)]) # 89 82 85 87 13 92 89 - numbers of classes for each chromosome
wnonzero_lengths <- lapply(wnonzero_hits_all, function(chr) sapply(chr, length)) # numbers of homopolymers on each chromosome for non empty classes only
names(wnonzero_hits_all) <- c("I","II","III","IV","MtDNA","V","X")

Generating GRanges object out of list of all motif hits:

sites.gr.worm <- lapply(1:7, function(i) do.call("c",lapply(lapply(wnonzero_hits_all[[i]], as, "IRanges"),
 GRanges,seqnames=names(wnonzero_hits_all)[i])))

Adding corresponding motif sequences and genomic homopolymer length to the GRanges object:

for (j in 1:7) {
 sites.gr.worm[[j]]$pattern.searched <- rep(as.character(pattern_all), sapply(whits_all[[j]], length))
 sites.gr.worm[[j]]$motif.found <- unlist(lapply(wnonzero_hits_all[[j]], as.character))
 sites.gr.worm[[j]]$pattern.length <- unlist(lapply(wnonzero_hits_all[[j]], width)) # includes the two flanking bases
 sites.gr.worm[[j]]$homopolymer.length <- (sites.gr.worm[[j]]$pattern.length)-2 # remove 2 bases, 5' and 3' are not part of homopolymer
}

Now we combine information from all chromosomes into one GRanges object showing all hits across the genome:

sites.gr_all.worm <- do.call("c",sites.gr.worm[-5]) # exclude MtDNA
genome(sites.gr_all.worm) <- "WBcel235" # add genome info

Creating a dataframe with homopolymer information:

homopolymers.worm <- as.data.frame(table(all_sites_worm$pattern.searched))
homopolymers.worm$length <- width(as.character(homopolymers.worm$Var1))-2 # include length of motif, -2 removes flanking 5'and 3' bases
homopolymers.worm$base <- ifelse(grepl("AAA",homopolymers.worm$Var1),"A",ifelse(grepl("TTT", homopolymers.worm$Var1), "T", ifelse(grepl("GGG", homopolymers.worm$Var1), "G", "C")))
homopolymer_frequency_by_length_worm <- homopolymers.worm %>%
 group_by(length) %>%
 summarise(total_frequency =sum(Freq))
homopolymer_frequency_by_length_worm$total_number_of_bases <- (homopolymer_frequency_by_length_worm$length)*as.numeric(homopolymer_frequency_by_length_worm$total_frequency) # calculate the number of bases for each HP

To identify *C. elegans* indels which intersect with homopolymers, we upload vcf from *C. elegans* samples (see Data Availability in the main text) with indels in a list of vcfs called MMRvcf.

# Select 1-bp indels
indel1bp <- sapply(MMRvcf, function(vcf) vcf[abs(width(granges(vcf)$REF)-width(unlist(granges(vcf)$ALT)))==1 &
 (width(granges(vcf)$REF)==1 | width(unlist(granges(vcf)$ALT))==1)])
barplot(sapply(indel1bp, length))

# Prepare the GRanges files
indel1bp <- sapply(indel1bp, rowRanges)
for (k in 1:length(indel1bp)) {
 indel1bp[[k]]$insertion <- width(unlist(indel1bp[[k]]$ALT))-1
 indel1bp[[k]]$deletion <- width(indel1bp[[k]]$REF)-1
}

Now we can check the intersections of homopolymers and indels; there can be cases when an indel is between 2 homopolymers and can be counted twice, so we will filter out the second occurrences of these variants.

worm_hit_list <- sapply(indel1bp, function(x) findOverlaps(x,sites.gr_all.worm))
indels.to.check.list <- sapply(worm_hit_list, function(x) unique(as.matrix(x)[,1])[which(table(as.matrix(x)[,1])>1)])
for (t in which(sapply(indels.to.check.list,length)>0)) {
 to.delete.final <- NULL
 indels.to.check <- indels.to.check.list[[t]]
 for (j in 1:length(indels.to.check)) {
 homopolymers.to.check <- as.matrix(worm_hit_list[[t]])[which(as.matrix(worm_hit_list[[t]])[,1] == (indels.to.check[j])),2]

 homopolymers.types <- sapply(sites.gr_all.worm[homopolymers.to.check]$pattern.searched,function(x) substr(x,2,2))

 ref <- unlist(strsplit(as.data.frame(indel1bp[[t]][indels.to.check[j]])$REF,split = ""))
 alt <- unlist(strsplit(as.character(as.data.frame(indel1bp[[t]][indels.to.check[j]])$ALT[[1]]),split = ""))

 if (indel1bp[[t]][indels.to.check[j]]$insertion==1) {
 indel.type <- setdiff(alt,ref)
 to.delete <- which(as.matrix(worm_hit_list[[t]])[,1]==indels.to.check[j]) [homopolymers.types != indel.type]
 if (length(to.delete)==0) {
 if (indel.type==alt[1]) to.delete <- which(as.matrix(worm_hit_list[[t]])[,1]==indels.to.check[j])[2]
 if (indel.type==alt[2]) to.delete <- which(as.matrix(worm_hit_list[[t]])[,1]==indels.to.check[j])[1]
 }
 }
 if (indel1bp[[t]][indels.to.check[j]]$deletion==1) {
 indel.type <- setdiff(ref,alt)
 to.delete <- which(as.matrix(worm_hit_list[[t]])[,1]==indels.to.check[j]) [homopolymers.types != indel.type]
 if (length(to.delete)==0) {
 if (indel.type==ref[1]) to.delete <- which(as.matrix(worm_hit_list[[t]])[,1]==indels.to.check[j])[2]
 if (indel.type==ref[2]) to.delete <- which(as.matrix(worm_hit_list[[t]])[,1]==indels.to.check[j])[1]
 }
 }
 to.delete.final <- c(to.delete.final,to.delete)
 }
 if (length(to.delete.final)>0) worm_hit_list[[t]] <- worm_hit_list[[t]][-to.delete.final]
}
worm_hit_list <- worm_hit_list[sapply(worm_hit_list,length)>0]

Making a list of data frames from the list of hits:

worm_hits_data_frame <- list()
for (i in 1:length(worm_hit_list)) {
 queries <- unique(queryHits(worm_hit_list[[i]]))
 idx <- sapply(queries, function(x) which(queryHits(worm_hit_list[[i]])==x)[1])
 idxs <- subjectHits(worm_hit_list[[i]])[idx]
 motifs <- DataFrame(sites.gr_all.worm$motif.found[idxs])
 length <- unlist(lapply(sites.gr_all.worm$motif.found[idxs], width))-2
 worm_hits_data_frame[[i]] <- as.data.frame(indel1bp[[names(worm_hit_list)[i]]][queries,])
 worm_hits_data_frame[[i]]$motif.found <- sites.gr_all.worm$motif.found[idxs]
 worm_hits_data_frame[[i]]$motif.length <- width(worm_hits_data_frame[[i]]$motif.found)
 worm_hits_data_frame[[i]]$homopolymer.length <- worm_hits_data_frame[[i]]$motif.length-2
 print(i)
}
names(worm_hits_data_frame) <- names(worm_hit_list)

Adding homopolymer type:

for (i in 1:length(worm_hits_data_frame)) {
 worm_hits_data_frame[[i]]$base <- ifelse(grepl("AAA",worm_hits_data_frame[[i]]$motif.found),"A",
 ifelse(grepl("TTT", worm_hits_data_frame[[i]]$motif.found), "T",
 ifelse(grepl("GGG", worm_hits_data_frame[[i]]$motif.found), "G", "C")))
 worm_hits_data_frame[[i]]$hpn <- paste0(worm_hits_data_frame[[i]]$homopolymer.length,worm_hits_data_frame[[i]]$base)
}

Grouping the data:

mlh1_20 = do.call("rbind",worm_hits_data_frame[c("CD0134a","CD0134c","CD0134d")])
pms2_20 = do.call("rbind",worm_hits_data_frame[c("CD0135a","CD0135c","CD0135d")])
pms2_10 = do.call("rbind",worm_hits_data_frame[c("CD0244a","CD0244c","CD0244d")])
pole4pms2_10 = do.call("rbind",worm_hits_data_frame[c("CD0246d","CD0246e")])
# Variant calling in long homopymers is infeasible (too few reads covering the whole homopolymer)
mlh1_20 = mlh1_20[mlh1_20$homopolymer.length<19,]
pms2_20 = pms2_20[pms2_20$homopolymer.length<19,]
pms2_10 = pms2_10[pms2_10$homopolymer.length<19,]
pole4pms2_10 = pole4pms2_10[pole4pms2_10$homopolymer.length<19,]

Plotting raw frequencies and ratios:

total_frequency = homopolymer_frequency_by_length_worm$total_frequency[1:15]
to.show <- data.frame(homopolymer.length = 4:(length(total_frequency)+3),
 pms2.20 = vector("numeric",length(total_frequency)),
 mlh1.20 = vector("numeric",length(total_frequency)),
 pms2.10 = vector("numeric",length(total_frequency)),
 pole4pms2.10 = vector("numeric",length(total_frequency)))
to.show$mlh1.20[as.numeric(names(table(mlh1_20$homopolymer.length)))-3] <- (table(mlh1_20$homopolymer.length)/3)
to.show$pms2.20[as.numeric(names(table(pms2_20$homopolymer.length)))-3] <- (table(pms2_20$homopolymer.length)/3)
to.show$pms2.10[as.numeric(names(table(pms2_10$homopolymer.length)))-3] <- (table(pms2_10$homopolymer.length)/3)
to.show$pole4pms2.10[as.numeric(names(table(pole4pms2_10$homopolymer.length)))-3] <- round(table(pole4pms2_10$homopolymer.length)/2)

Plotting absolute numbers:

df <- melt(to.show,id.vars = "homopolymer.length")
ggplot(data=df,aes(x=homopolymer.length,y=value)) + geom_bar(stat='identity', width = 0.75) +
 facet_grid(. ~ factor(variable,levels=c("mlh1.20","pms2.20","pms2.10","pole4pms2.10"))) +theme_bw() +
 ylab("Number of 1 bp indels") + xlab("Homopolymer length") +
 theme(axis.text = element_text(size=12, family='ArialMT'),
 axis.title = element_text(size=16, family='ArialMT'),
 strip.text = element_text(size=16, family='ArialMT'),
 panel.grid = element_blank(), strip.background = element_blank())

Plotting relative frequencies:

for (i in 2:ncol(to.show)) to.show[,i] = to.show[,i] / total_frequency
df <- melt(to.show,id.vars = "homopolymer.length")
ggplot(data=df,aes(x=homopolymer.length,y=value)) +
 facet_grid(. ~ factor(variable,levels=c("mlh1.20","pms2.20","pms2.10","pole4pms2.10"))) +
 geom_point() + geom_line() + theme_bw() +
 ylab("Ratio \n Mutations/HP") + xlab("Homopolymer length") +
 theme(axis.text = element_text(size=12), axis.title = element_text(size=16), strip.text = element_text(size=16))

Next, we apply a generalized additive model with splines to smooth the relative frequency, and plot the results:

total_frequency = homopolymer_frequency_by_length_worm$total_frequency[1:15]
to.show <- data.frame(homopolymer.length = 4:(length(total_frequency)+3),
 pms2 = vector("numeric",length(total_frequency)),
 mlh1= vector("numeric",length(total_frequency)),
 pole4pms2= vector("numeric",length(total_frequency)),
 pms210 = vector("numeric",length(total_frequency)),
 total_frequency = total_frequency,
 total_number_of_bases = homopolymer_frequency_by_length_worm$total_number_of_bases[1:15])
to.show$mlh1[as.numeric(names(table(mlh1_20$homopolymer.length)))-3] <- round(table(mlh1_20$homopolymer.length)/3)
to.show$pms2[as.numeric(names(table(pms2_20$homopolymer.length)))-3] <- round(table(pms2_20$homopolymer.length)/3)
to.show$pms210[as.numeric(names(table(pms2_10$homopolymer.length)))-3] <- round(table(pms2_10$homopolymer.length)/3)
to.show$pole4pms2[as.numeric(names(table(pole4pms2_10$homopolymer.length)))-3] <- round(table(pole4pms2_10$homopolymer.length)/2)

*mlh-1* mutants, generation 20

fit <- gam(cbind(mlh1, total_frequency-mlh1) ~ s(homopolymer.length, 6), data=to.show, family=binomial)
plot(to.show$homopolymer.length, to.show$mlh1/to.show$total_frequency, xlim=c(4,18), ylim=c(0,0.015), ylab="Ratio \n Mutations/HP", pch=16, col='grey', main='mlh-1',font.main=3, xlab="Homopolymer length")
segments(to.show$homopolymer.length, qbeta(0.025, .5+to.show$mlh1, .5+to.show$total_frequency- to.show$mlh1),
 to.show$homopolymer.length, qbeta(0.975, .5+to.show$mlh1, .5+to.show$total_frequency- to.show$mlh1), col='grey')
p <- predict(fit, type='link', se.fit=TRUE)
lines(to.show$homopolymer.length, exp(p$fit), col='red', lwd=2)
lines(to.show$homopolymer.length, exp( p$fit + 2*p$se.fit), col='red', lty=2)
lines(to.show$homopolymer.length, exp(p$fit - 2* p$se.fit), col='red', lty=2)
lines(to.show$homopolymer.length, exp(p$fit), col='#FF00000d', lwd=2)
for(q in seq(0.025, 0.475,0.025))
 polygon(c(to.show$homopolymer.length, rev(to.show$homopolymer.length)), c(exp(qnorm(q, p$fit, p$se.fit)), exp(rev(qnorm(1-q, p$fit, p$se.fit)))), border=NA, col='#FF00000d')

*pms-2* mutants, generation 20

fit <- gam(cbind(pms2, total_frequency-pms2) ~ s(homopolymer.length, 6), data=to.show, family=binomial)
plot(to.show$homopolymer.length, to.show$pms2/to.show$total_frequency, yaxt="n",
 xlim=c(4,18), ylim=c(0,0.015), ylab=NA,
 pch=16, col='grey', main='pms-2',font.main=3, xlab="Homopolymer length")
segments(to.show$homopolymer.length, qbeta(0.025, .5+to.show$pms2, .5+to.show$total_frequency- to.show$pms2),
 to.show$homopolymer.length, qbeta(0.975, .5+to.show$pms2, .5+to.show$total_frequency- to.show$pms2), col='grey')
p <- predict(fit, type='link', se.fit=TRUE)
lines(to.show$homopolymer.length, exp(p$fit), col='red', lwd=2)
lines(to.show$homopolymer.length, exp( p$fit + 2*p$se.fit), col='red', lty=2)
lines(to.show$homopolymer.length, exp(p$fit - 2* p$se.fit), col='red', lty=2)
lines(to.show$homopolymer.length, exp(p$fit), col='#FF00000d', lwd=2)
for(q in seq(0.025, 0.475,0.025))
 polygon(c(to.show$homopolymer.length, rev(to.show$homopolymer.length)), c(exp(qnorm(q, p$fit, p$se.fit)), exp(rev(qnorm(1-q, p$fit, p$se.fit)))), border=NA, col='#FF00000d')

*pms-2* mutants, generation 10

fit <- gam(cbind(pms210, total_frequency-pms210) ~ s(homopolymer.length, 6), data=to.show, family=binomial)
plot(to.show$homopolymer.length, to.show$pms210/to.show$total_frequency, yaxt="n", xlim=c(4,18), ylim=c(0,0.015), ylab=NA, pch=16, col='grey', main='pms-2 10',font.main=3, xlab="Homopolymer length")
segments(to.show$homopolymer.length, qbeta(0.025, .5+to.show$pms210, .5+to.show$total_frequency- to.show$pms210),
 to.show$homopolymer.length, qbeta(0.975, .5+to.show$pms210, .5+to.show$total_frequency- to.show$pms210), col='grey')
p <- predict(fit, type='link', se.fit=TRUE)
lines(to.show$homopolymer.length, exp(p$fit), col='red', lwd=2)
lines(to.show$homopolymer.length, exp( p$fit + 2*p$se.fit), col='red', lty=2)
lines(to.show$homopolymer.length, exp(p$fit - 2* p$se.fit), col='red', lty=2)
lines(to.show$homopolymer.length, exp(p$fit), col='#FF00000d', lwd=2)
for(q in seq(0.025, 0.475,0.025))
 polygon(c(to.show$homopolymer.length, rev(to.show$homopolymer.length)), c(exp(qnorm(q, p$fit, p$se.fit)), exp(rev(qnorm(1-q, p$fit, p$se.fit)))), border=NA, col='#FF00000d')

Double mutants, *pole-4; pms-2*, generation 10

fit <- gam(cbind(pole4pms2, total_frequency-pole4pms2) ~ s(homopolymer.length, 6), data=to.show, family=binomial)
plot(to.show$homopolymer.length, to.show$pole4pms2/to.show$total_frequency, yaxt="n", xlim=c(4,18), ylim=c(0,0.015), ylab=NA, pch=16, col='grey', main='pole-4; pms-2',font.main=3, xlab="Homopolymer length")
segments(to.show$homopolymer.length, qbeta(0.025, .5+to.show$pole4pms2, .5+to.show$total_frequency- to.show$pole4pms2),
 to.show$homopolymer.length, qbeta(0.975, .5+to.show$pole4pms2, .5+to.show$total_frequency- to.show$pole4pms2), col='grey')
p <- predict(fit, type='link', se.fit=TRUE)
lines(to.show$homopolymer.length, exp(p$fit), col='red', lwd=2)
lines(to.show$homopolymer.length, exp( p$fit + 2*p$se.fit), col='red', lty=2)
lines(to.show$homopolymer.length, exp(p$fit - 2* p$se.fit), col='red', lty=2)
lines(to.show$homopolymer.length, exp(p$fit), col='#FF00000d', lwd=2)
for(q in seq(0.025, 0.475,0.025))
 polygon(c(to.show$homopolymer.length, rev(to.show$homopolymer.length)), c(exp(qnorm(q, p$fit, p$se.fit)), exp(rev(qnorm(1-q, p$fit, p$se.fit)))), border=NA, col='#FF00000d')
