# Supplementary material for: Mutational signatures of DNA mismatch repair deficiency in C. elegans and human cancers
Source: Genome Res. 2018 May;28(5):666–75. doi: 10.1101/gr.226845.117 (PMC5932607; doi:10.1101/gr.226845.117)
Supplement: Supplemental Material [file supp_gr.226845.117_Supplemental_Material.docx]

SUPPLEMENTAL MATERIAL

***C. elegans* strains, crosses, maintenance and propagation.**

To obtain the *pole-4 II; pms-2 V* double mutant, freshly backcrossed *pole-4(tm4613)* and *pms-2(ok2529)* lines were crossed to each other and F2 individuals were genotyped to obtain the four genotypes N2 wild-type (TG3551), *pms-2* (TG3552), *pole-4* (TG3553) and *pole-4; pms-2* (TG3554). Backcrossed strains were frozen for glycerol stocks. 5 to 10 lines were propagated per genotype for mutation accumulation over 10 or 20 generations. Genomic DNA was isolated from the initial (P0 or F1) and two to three lines of the final generation per genotype using ChargeSwitch® gDNA Mini Tissue Kit (Invitrogen) and used for whole-genome sequencing as described (Meier et al. 2014).

**Post-processing of mutation calls**

Variant calling and post-processing filters were applied as described previously (Meier et al. 2014) with the following modifications.

*For substitutions*:

4. Maximum number of reads reporting the variant base in the ‘control’ worm = 1;

5. Maximum number of other samples variant called in = 0;

7. No more than one read mapped with an indel across the base;

8. Variant dropped if any one sample in panel of 5 ‘control’ worms has variant in 2 or more reads and 5% or more total reads.

*For indels*:

1. No filter was used limiting the number of units at site;

2. More than 20% reads in 'test worm' reporting variant.

**Reliability of indel calls in homopolymer sequences in *C. elegans***

Small indels in repetitive sequences could be generated as polymerase errors during bridge amplification or sequencing, leading to possible sequencing artifacts. Across a total of 101 wild-type samples of different generations (including those shown in Fig. 1C), 7433 1bp indels were observed on average prior to post processing. Of these, 7109 1bp indels on average occurred in homopolymer runs. Following filtering (see “Post-processing of mutation calls”) we observed on average 0.5 of 0.54 1bp indels arising in homopolymers per sample, with the majority of indels being removed when filtering for quality and frequency of mutant reads (see “Post-processing of mutation calls”). Thus 1bp indels likely arising during PCR amplification or sequencing seem to be efficiently removed using our filtering procedure.

**Selection of the number of signatures during *de novo* signature extraction**

The number of signatures was chosen based on the saturation of both the Akaike Information Criterion (AIC) (Akaike 1973) and the residual sum of squares (RSS). The AIC is calculated as $AIC=2k\cdot(n+N)-2logL$, where *k*￼ is the number of parameters (with *n* being the number of signatures and *N* being the number of samples), and *L* is the maximized model likelihood. As the *L* would naturally increase with the addition of parameters, we performed signature extraction for different ranks and chose the one where AIC and also RSS decrease slows down to avoid oversegmentation (Supplemental Fig. S3A).

**Analysis of the SNP contaminated *de novo* signature**

The presence of a high number of SNPs in the *de novo* signature classified as “SNP-contaminated”, was detected by overlapping the base substitutions from the samples with high prevalence of this signature (relative contribution over 50%) with SNPs prevalent in the human population (dbSNP Build 144 database, (Pagès 2017). The fraction of SNP intersection for these samples was 0.76 on average (IQR 0.76-0.85) compared to 0.18 (IQR 0-0.4) for other samples. Moreover, information of coding and non-coding SNPs (1000 Genomes database, <http://www.internationalgenome.org/home>) was used to calculate the non-synonymous to synonymous change ratio across these samples, which was lower than expected for cancer variants (0.8 on average, IQR 0.68-0.88, whereas cancer variants are expected to show a ratio higher than 1), suggesting that these substitutions represent germline variants.

**Mutations in MMR-related genes**

Analysis of mutations in MMR-related genes MLH1, PMS2, MSH2, MSH3, MSH6, PMS1, MLH3 and EPCAM (Hsieh and Yamane 2008; Ligtenberg et al. 2009) consisted of identification of high-impact mutations (missense, frameshift or splice region variants, disruptive in-frame deletions or insertions, stop gained, start lost, and stop lost) in the genes of interest. Hypermethylation of MLH1 was defined by correlating the methylation values of 43 CpG sites in the CpG island overlapping the MLH1 gene promoter with MLH1 expression level. Expression and methylation data for this analysis were obtained from ICGC for COAD and STAD samples (http://icgc.org).

**Calculation of mutational patterns of individual factors in *C. elegans* samples**

To extract the signatures of individual factors (single genes and interaction term) from respective *C. elegans* samples, we used additive Poisson model with multiple factors for every trinucleotide context and indel type (104 in total):

$$Y_{j}\sim\mathrm{Pois}\left( \lambda_{j} \right), j=1, \ldots, 104$$

$$E\left[ Y_{j} \right]=\lambda_{j}=N\left( {\beta_{j,b}+ X}_{g_{1}}{\beta_{j,g_{1}}+ X}_{g_{2}}\beta_{j,g_{2}}+X_{g_{1}:g_{2}}\beta_{j,g_{1}:g_{2}} \right) ,$$

$$\beta_{j,\cdot}\geq0,$$

where $Y_{j}$ is a number of particular mutations happening in a given context (out of 104 types), *N* is the number of generations, and $g1$ and $g2$ – gene knock-outs, $b$ - background contribution, and coefficient $X_{\cdots}\in\{0,1\}$ indicates the presence of a particular factor.

We calculated maximum likelihood estimates of all 104-long vectors $\beta_{j, \cdot}$ for every signature using an algorithm equivalent to non-negative matrix factorization with a fixed multiplier and KL-divergence as distance measure (Lee and Seung 2000; Cemgil 2009). 95% confidence intervals for signatures were calculated as CIs for Poisson regression coefficients.

**Mutational signature comparison using cosine similarities**

The threshold for ‘high’ similarity was taken as 0.80, as the similarity assessment within the COSMIC cancer signature set showed that only 3.4% of pairs have a cosine similarity above this threshold; it also demarcates the 95% quantile of the similarity distribution between random uniformly generated ‘mutational profile’ vectors from the positive cone (Supplemental Fig. S6A, B).

Due to statistical challenges of separating correlated processes (such as MMR deficiency and 5meC deamination), we measured the uncertainty of signature comparison. Stability assessment of the similarity score between *C. elegans* signatures and MMR-1 was performed by calculating similarities of random draws from *C. elegans* signatures' 95% confidence intervals and the signature most similar to MMR-1 among the ones calculated for each jack-knife (leave-one-out) bootstrapped sample of the COAD/STAD dataset (Supplemental Fig. S6C, D).

Given that the cancer types we analyzed (COAD and STAD projects) were reported to contain different sets of COSMIC MMR-associated signatures (http://cancer.sanger.ac.uk/cosmic/signatures), we also assessed the similarity of aggregated profiles of MMR-deficient samples between the cohorts. Average mutational profiles were extremely similar (similarity score of 0.99), with most of the variability coming from different contributions of C>T mutations in an NCG context (Supplemental Fig. S5C).

**Supplemental References**

Akaike H. 1973. Information Theory and an Extension of the Maximum Likelihood Principle. *2nd International Symposium of Information Theory, Akademiai Kiado, Budapest*: 267-281.

Cemgil AT. 2009. Bayesian Inference for Nonnegative Matrix Factorisation Models. *Computational Intelligence and Neuroscience* **2009**: 17 pages.

Hsieh P, Yamane K. 2008. DNA mismatch repair: molecular mechanism, cancer, and ageing. *Mech Ageing Dev* **129**: 391-407.

Lee DD, Seung HS. 2000. Algorithms for Non-negative Matrix Factorization. *Advances in Neural Information Processing* **13**.

Ligtenberg MJ, Kuiper RP, Chan TL, Goossens M, Hebeda KM, Voorendt M, Lee TY, Bodmer D, Hoenselaar E, Hendriks-Cornelissen SJ et al. 2009. Heritable somatic methylation and inactivation of MSH2 in families with Lynch syndrome due to deletion of the 3' exons of TACSTD1. *Nature genetics* **41**: 112-117.

Meier B, Cooke SL, Weiss J, Bailly AP, Alexandrov LB, Marshall J, Raine K, Maddison M, Anderson E, Stratton MR et al. 2014. C. elegans whole-genome sequencing reveals mutational signatures related to carcinogens and DNA repair deficiency. *Genome research* **24**: 1624-1636.

Pagès H. 2017. SNPlocs.Hsapiens.dbSNP141.GRCh38: SNP locations for Homo sapiens (dbSNP Build 141). *R package version* **0.99.11**.

Robinson JT, Thorvaldsdottir H, Winckler W, Guttman M, Lander ES, Getz G, Mesirov JP. 2011. Integrative genomics viewer. *Nature biotechnology* **29**: 24-26.

Thorvaldsdottir H, Robinson JT, Mesirov JP. 2013. Integrative Genomics Viewer (IGV): high-performance genomics data visualization and exploration. *Briefings in bioinformatics* **14**: 178-192.

SUPPLEMENTAL FIGURES

**Supplemental Figure S1.** **Base substitutions observed in repetitive regions in *pms‑2* mutants grown for 10 generations.** **(A)** Representative sequence alignments depicting T>A and A>T mutations as identified in Figure 1C in a larger sequence context. Shown are sequence reads aligned to the reference genome WBcel235.74 and visualized in Integrative Genomics Viewer (IGV) (Robinson et al. 2011; Thorvaldsdottir et al. 2013). Wild-type DNA sequence is displayed at the bottom of each panel and base substitutions are indicated as mutated to A, T, C, and G in green, red, blue, and brown, respectively. **(B-D)** IGV representation of sequence read alignments across rare events of 2-4 base substitution clusters with mutations that change the underlying sequence to be more similar or identical to an adjacent repeat region. Grey bars above the colored reads indicate read coverage. Mutated bases are indicated as colored bases in each sequence read in which they are present. Schemes below alignments show the larger sequence context and its repetitive nature. Differences between wild-type and mutant lines are highlighted.

**Supplemental Figure S2.** **Distribution of mutations observed in *pms-2* and *pole-4; pms-2* mutants grown for 10 generations across the six *C. elegans* chromosomes**. **(A)** Genomic position of all unique base substitutions detected across three *pms-2* and two *pole-4; pms-2* F10 lines plotted by chromosome. Bases mutated are indicated in color as shown in the legend; grey bars indicate the respective chromosome length. **(B)** Genomic distribution of all homopolymer runs within the *C. elegans* genome (top panel) and all unique indels detected across three *pms-2* (bottom left) and two *pole-4; pms-2* (bottom right) lines by chromosome. Shades of blue (top panel) indicate the frequency of homopolymers within a given chromosomal interval. Blue dots (bottom panels) indicate the chromosomal position of individual indels.

**Supplemental Figure S3. Criteria for signature extraction from whole exome variant calls from combined colorectal cancer (COAD) and gastric cancer (STAD) datasets and signature comparison to their most related COSMIC signatures. (A)** AIC (Akaike information criterion) and RSS (residual sum of squares) comparison to determine the relative quality of models on the combined COAD and STAD dataset for signature extraction with different ranks. The dashed red line denotes the measure level for a model with 8 signatures chosen as the optimal value. **(B)** Comparison of *de novo* mutational signatures extracted from combined COAD and STAD whole exome datasets and their most similar exome-adjusted COSMIC signatures: Clock-1 and COSMIC signature 1 (0.98 similarity), Clock-2 and COSMIC signatures 2, 3 and 5 (similarities 0.62, 0.75 and 0.66, respectively), POLE and COSMIC signature 10 (0.97 similarity), “17-like” and COSMIC signature 17 (0.98 similarity), MMR-1 and COSMIC signature 20 (0.85 similarity), MMR-2 and COSMIC signature 15 (0.99 similarity), MMR-3 and COSMIC signatures 21 and 26 (0.95 and 0.91 similarity, respectively), SNP and COSMIC signatures 5 and 20 (0.83 and 0.79 similarity, respectively).

**Supplemental Figure S4. Distribution of homopolymers in the human exome and numbers of 1 bp indels in homopolymers for COAD and STAD samples with MSI. (A)** Homopolymer numbers and the fractions of homopolymers by base in the human exome. The base distribution of homopolymers in the human exome is close to uniform, and differs from the homopolymer distribution in the *C. elegans* (94% AT) and the human (78% AT) genome. **(B)** Numbers of 1 bp indels in homopolymers (HP) and other sequence contexts for COAD (top) and STAD (bottom) MSI (microsatellite instable-high) samples. On average 69-72% and 91-93% of 1 bp indels occur in homopolymers in samples from the COAD and the STAD cohort, respectively.

**Supplemental Figure S5. Decomposition of mutational profiles in colorectal and stomach cancer cohorts over *de novo* extracted signatures. (A)** Decomposition of mutational profiles of COAD samples over eight *de novo* extracted signatures. The main plot shows absolute contributions of each signature to mutation numbers for all samples of the COAD cohort. The center panel represents relative signature contributions in COAD samples with microsatellite instability (MSI samples). MSI samples are clearly denoted by the presence of signature MMR-1 (green), with several samples showing high contributions of MMR-2 and MMR-3 signatures (purple and orange, respectively). **(B)** Decomposition of mutational profiles of STAD samples over eight *de novo* extracted signatures. The main plot shows absolute contributions of each signature to mutation numbers for all samples of the STAD cohort. The center panel represents relative signature contributions for MSI samples. **(C)** Per-substitution type per-context variability of mutation profiles from MSI samples in COAD (top) and STAD (bottom) datasets. The averaged profiles look extremely similar (0.99 similarity between the per-type per-context means) with most of the variability coming from different fractions of C>T transitions in NCG contexts.

**Supplemental Figure S6. Similarity analysis between mutational signatures.**

**(A)** Frequency of cosine similarity scores for all pairwise signature comparisons across the 30 COSMIC cancer signatures. 3% of signature pairs exhibited a similarity higher than 0.80, a threshold indicated by a red dashed line. **(B)** Cosine similarity distribution for random vectors drawn uniformly from 0 to y_max (individual for every draw). The 0.95-quantile of this distribution is 0.80 indicated by a red dashed line. **(C)** Distribution of similarities between random draws from 95% confidence intervals for *C. elegans* mutational patterns: *mlh-1* versus *pole-4; pms-2* (top panel), *pms-2* versus *pole-4; pms-2* (center panel), *pms-2* versus *mlh-1* (bottom panel). Red dashed lines represent the similarity values for fixed initial patterns (0.70 for *mlh-1* versus *pole4; pms-2*, 0.71 for *pms-2* versus *pole-4; pms-2*, 0.96 for *mlh-1* versus *pms‑2*) (Supplemental Material). **(D)** Distribution of similarities between the MMR-1 signature from bootstrapped samples of the COAD/STAD dataset and random draw from 95%-confidence intervals for *mlh-1* (top panel) and *pms-2* (lower panel) mutational patterns. Red dashed lines represent the similarity values for fixed initial signatures: 0.77 (resulting inter-quantile range (IQR) 0.73 to 0.80) and 0.90 (IQR 0.90-0.91) for *mlh-1,* 0.81 (IQR 0.77 to 0.85) and 0.92 (IQR 0.92-0.93) for *pms-2* with and without C>T at CpG sites, respectively.

**Supplemental Figure S7.** **Comparison of humanized mutation patterns from *C. elegans* *pms-2*, *mlh-1* single and *pole-4; pms-2* double mutants with MMR-associated COSMIC signatures.** Learned mutational patterns from *C. elegans* *pms‑2*, *mlh-1* single and *pole-4; pms-2* double mutants adjusted for the difference in trinucleotide frequency between the *C. elegans* genome and human exome, and COSMIC signatures 6, 15, 20 and 26, previously associated with DNA mismatch repair deficiency (Supplemental Material). All signatures are shown as whole exome adjusted versions. Green and red stars indicate C>T transitions at CpG sites, which occur at lower frequency in *C. elegans*.
